# Specific Sn–O–Fe Active Sites from Atomically Sn-Doping Porous Fe_2_O_3_ for Ultrasensitive NO_2_ Detection

**DOI:** 10.1007/s40820-025-01770-9

**Published:** 2025-05-26

**Authors:** Yihong Zhong, Guotao Yuan, Dequan Bao, Yi Tao, Zhenqiu Gao, Wei Zhao, Shuo Li, Yuting Yang, Pingping Zhang, Hao Zhang, Xuhui Sun

**Affiliations:** 1 Institute of Functional Nano and Soft Materials (FUNSOM), Jiangsu Key Laboratory for Carbon-Based Functional Materials and Devices Jiangsu Key Laboratory of Advanced Negative Carbon Technologies, Soochow University, Suzhou, 215123 People’s Republic of China; 2 College of Chemistry and Environmental Engineering, Shenzhen University, Shenzhen, 518060 People’s Republic of China; 3 The Key Laboratory of Rare Earth Functional Materials and Applications, Zhoukou Normal University, Zhoukou, 466000 People’s Republic of China; 4 Suzhou Huiwen Nanotechnology Co., Ltd., Suzhou, 215000 People’s Republic of China; 5 Materials Science Gusu Laboratory, Suzhou, 215123 China

**Keywords:** Atomically doping, Specific Sn–O–Fe sites, NO_2_ detection, Gas sensor, Specific adsorption

## Abstract

Conventional gas sensing materials (e.g., metal oxides) suffer from deficient sensitivity and serve cross-sensitivity issues due to the lack of efficient adsorption sites. Herein, the heteroatom atomically doping strategy is demonstrated to significantly enhance the sensing performance of metal oxides-based gas sensing materials. Specifically, the Sn atoms were incorporated into porous Fe_2_O_3_ in the form of atomically dispersed sites. As revealed by X-ray absorption spectroscopy and atomic-resolution scanning transmission electron microscopy, these Sn atoms successfully occupy the Fe sites in the Fe_2_O_3_ lattice, forming the unique Sn–O–Fe sites. Compared to Fe–O–Fe sites (from bare Fe_2_O_3_) and Sn–O–Sn sites (from SnO_2_/Fe_2_O_3_ with high Sn loading), the Sn–O–Fe sites on porous Fe_2_O_3_ exhibit a superior sensitivity (*R*_g_/*R*_a_ = 2646.6) to 1 ppm NO_2_, along with dramatically increased selectivity and ultra-low limits of detection (10 ppb). Further theoretical calculations suggest that the strong adsorption of NO_2_ on Sn–O–Fe sites (N atom on Sn site, O atom on Fe site) contributes a more efficient gas response, compared to NO_2_ on Fe–O–Fe sites and other gases on Sn–O–Fe sites. Moreover, the incorporated Sn atoms reduce the bandgap of Fe_2_O_3_, not only facilitating the electron release but also increasing the NO_2_ adsorption at a low working temperature (150 °C). This work introduces an effective strategy to construct effective adsorption sites that show a unique response to specific gas molecules, potentially promoting the rational design of atomically modified gas sensing materials with high sensitivity and high selectivity.

**Supplementary Information:**

The online version contains supplementary material available at 10.1007/s40820-025-01770-9.

## Highlights

- The heteroatom atomically doping strategy was reported to construct highly efficient sites on metal oxides for the detection of low-concentration gas.
- The atomically dispersed Sn atoms were intentionally incorporated into the Fe_2_O_3_ lattice during the oxidative annealing of Fe-based metal organic framework, leading to specific Sn–O–Fe sites, porous structures, and abundant oxygen vacancies.
- The optimized Sn-Fe_2_O_3_ exhibited exceptional sensing performance for NO_2_ detection: ultra-high sensitivity (*Rg*/*Ra*=2646.6 to 1 ppm NO_2_), ultra-low limit of detection (10 ppb), and high selectivity.

**Supplementary Information:**

The online version contains supplementary material available at 10.1007/s40820-025-01770-9.

## Graphical abstract

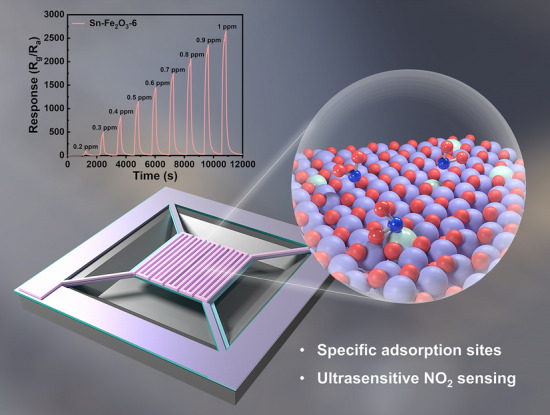

## Introduction

In contemporary industrial production and residential life, the timely monitoring of particular gases is of paramount importance [1–6]. As one of the most hazardous gases, nitrogen dioxide (NO_2_) could cause serious respiratory diseases even in a trace amount [7, 8]. The World Health Organization recommends that NO_2_ concentration in the environment should not exceed 82 ppb, highlighting the importance of monitoring low-concentration NO_2_ [9]. Generally, metal oxide semiconductors (MOS) have been widely used as gas sensing material owing to their comprehensive features, such as semiconducting character, nontoxicity, abundance, and chemical stability [10, 11]. However, most MOS-based gas sensors for NO_2_ detection have to operate at a high temperature (> 200 °C) and exhibit limited response and selectivity [12]. The unsatisfactory performance is mainly due to the lack of effective adsorption sites on metal oxides, and the cross-sensitivity issues would become more severe for low-concentration gas detection. Therefore, constructing highly efficient adsorption sites on MOS is of great significance to realize the detection of low-concentration NO_2_.

Recently, single-atom catalysts (SACs) featuring maximum atomic efficiency have attracted extensive attention in (electro)catalysis fields, in which the atomically dispersed sites with specific active structures present unprecedented catalytic performance [13–17]. Compared to nanoparticles or nanoclusters, SAC can maximally expose the active centers to boost the reaction efficiency [18]. Considering that the gas sensing process resembles surface catalysis, the atomically dispersed sites from SACs are expected to offer excellent gas sensing performance. For example, Gu et al. prepared Pt SAC on WO_3_ using the template method, in which the atomically dispersed Pt with high activity showed exceptional response toward triethylamine [19]. Feng et al. designed the SnO_2_ nanospheres functionalized by Au SAC, resulting in a Listeria monocytogene sensor with high sensitivity and selectivity [20]. In addition, Wang et al. constructed Cu SAC as catalytic sites on WO_2.72_ nanowires (Cu SAC/WO_2.72_) for the detection of toluene [21]. The atomically dispersed Cu sites contributed to a strong affinity toward toluene and a lowered reaction barrier. The above works demonstrate that the atomically dispersed sites can significantly enhance the gas sensing performance of MOS-based gas sensors [22, 23]. Notably, directly incorporating atomically dispersed sites onto the surface of metal oxides might produce various active sites with multiple coordination environments, unavoidably altering the adsorption strength of gas molecules and reducing the sensing selectivity of corresponding sensors. Therefore, precisely controlling the location of atomically dispersed sites in metal oxides is essential to ensuring the structural uniformity of exposed active sites but remains challenging.

In this work, atomically dispersed Sn atoms were incorporated into the Fe_2_O_3_ lattice, which offers highly efficient sites for the detection of low-concentration NO_2_. Firstly, the ligands in Fe-based metal organic framework (MOF) are utilized as the fences to spatially separate these Sn atoms. During the following annealing process, Fe-based MOF transforms into porous Fe_2_O_3_; meanwhile, Sn atoms enter into the Fe_2_O_3_ lattice and occupy the lattice sites of Fe atoms, leading to the formation of Sn–O–Fe sites. The specific coordination environments of the Sn–O–Fe sites have been verified by high-angle annular dark-field scanning transmission electron microscopy (HAADF-STEM) and synchrotron-based X-ray absorption spectroscopy (XAS) measurements. The incorporated Sn atoms not only regulate the energy band structure of α-Fe_2_O_3_ but also enhance specific adsorption to NO_2_ molecules. Additionally, the obtained Fe_2_O_3_ possesses a mesoporous structure and abundant oxygen vacancies, both of which are conducive to the gas sensing process. As a result, the optimized sample (Sn–Fe_2_O_3_–6) exhibits extraordinary sensing response to NO_2_ (*R* = 2646.6 to 1 ppm NO_2_ at 150 °C), ultra-low detection limit (10 ppb), and excellent selectivity.

## Experimental Section

### Reagents and Materials

Pluronic F-127 (EO_97_PO_69_EO_97_, with an average *M*_n_ = 12,600) is obtained from Sigma-Aldrich. Iron chloride hexahydrate (FeCl_3_·6H_2_O, 99%), stannic chloride pentahydrate (SnCl_4_·5H_2_O, 99%), 2-aminoterephthalic acid (H_2_N-BDC, 99%), and acetic acid (CH_3_COOH, 99.7%) were obtained Shanghai Aladdin Biochemical Technology Co., Ltd. All chemicals were used as received without undergoing an additional purification process.

### Synthesis of Sn-Fe_2_O_3_

#### Fe-MIL-88B-NH_2_-Sn-X Precursors

The Fe-MOF was prepared by a hydrothermal method based on the previous work with some modifications [24]. Typically, 0.32 g of Pluronic F-127 was dissolved in 26.7 mL of deionized water and stirred for 2 h. Next, 3.3 mL of FeCl_3_⋅6H_2_O (0.4 M) was added to the surfactant solution. The resulting mixture was stirred for 1.5 h before injecting 0.6 mL of acetic acid. After stirring for 1.5 h, 120 mg of 2-aminoterephthalic acid was added and stirred for an additional 2 h. The resulting resolution was then transferred into a 50 mL Teflon-lined stainless steel autoclave and heated at 110 °C for 24 h. After cooling to room temperature, the dark brown powders were collected by centrifugation and purified with ethanol three times. Finally, the as-synthesized Fe-MOF denoted as Fe-MIL-88B-NH_2_-Sn-0 was dried overnight in a vacuum dryer at 60 °C. The Fe-MIL-88B-NH_2_-Sn-X (*X* = 2, 4, 6, 8) was synthesized using the same method as before, with the addition of SnCl_4_·5H_2_O to the surfactant solution while adding FeCl_3_·6H_2_O. The molar ratios of Sn ions to Fe ions were set as 2%, 4%, 6%, and 8%, denoted as Fe-MIL-88B-NH_2_-Sn-2, Fe-MIL-88B-NH_2_-Sn-4, MIL-88B-NH_2_-Sn-6, and Fe-MIL-88B-NH_2_-Sn-8, respectively.

#### Sn-Fe_2_O_3_-X

MOF-derived Sn-Fe_2_O_3_-X (*X* = 0, 2, 4, 6, 8) were obtained by annealing the Fe-MIL-88B-NH_2_-Sn-X at 500 °C for 120 min with a heating rate of 2 °C min^−1^ in a muffle furnace under an air atmosphere.

#### Gas Sensing Performance

Alumina substrate gas sensor and MEMS gas sensor were used for gas sensing testing. Detailed experimental procedures and methods are exhibited in the Supporting Information.

## Results and Discussion

### Synthesis and Characterizations of Sn-Fe_2_O_3_-X

**Fig. 1:**
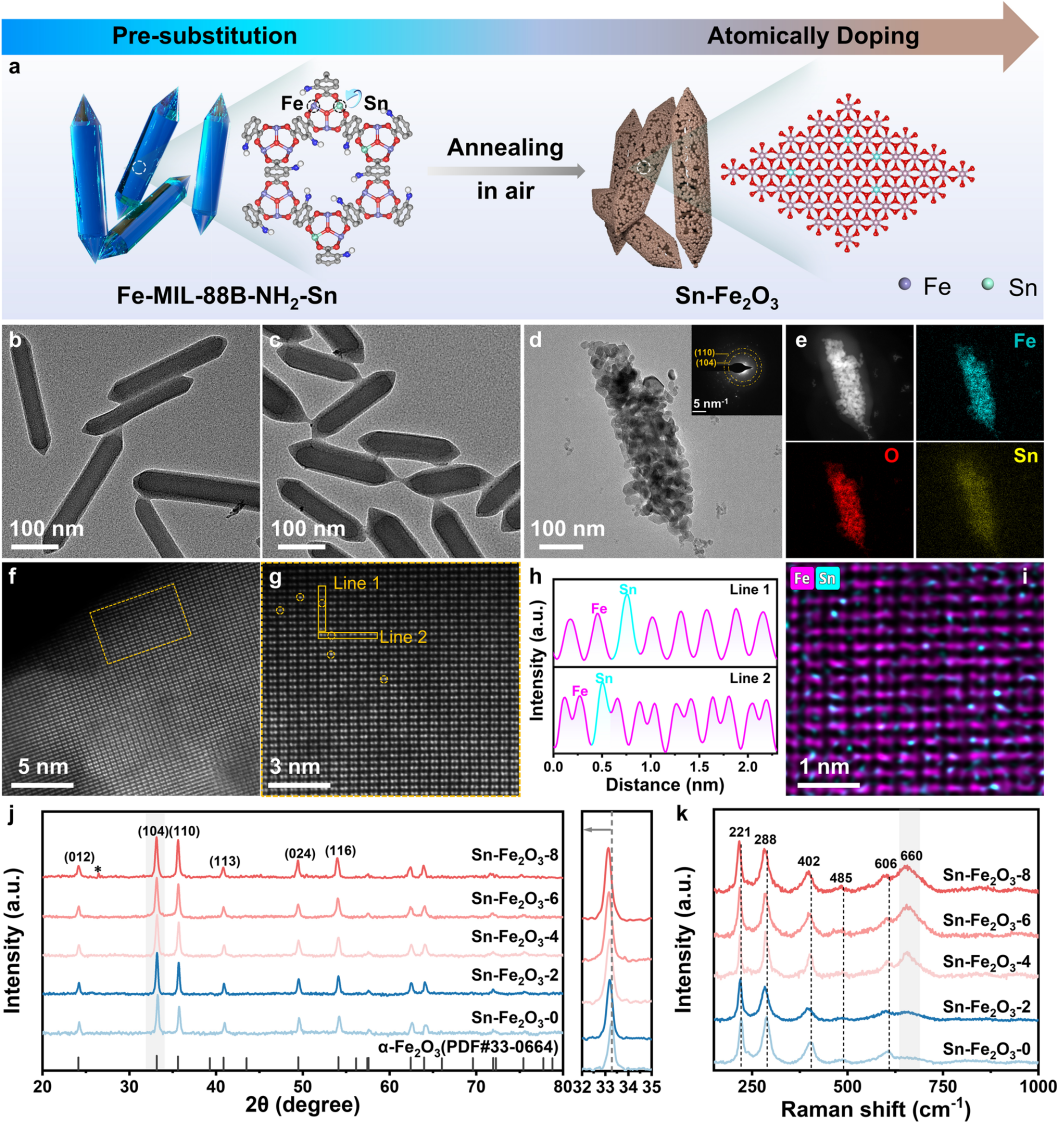
Characterization of Sn-Fe_2_O_3_-X. **a** Schematic of synthesis procedures to atomically doping Sn sites in α-Fe_2_O_3_. TEM images of **b** Fe-MIL-88B-NH_2_-Sn-0, **c** Fe-MIL-88B-NH_2_-Sn-6. **d** TEM image of Sn-Fe_2_O_3_-6, along with SAED pattern (inset) and corresponding **e** HADDF-STEM image and EDX elemental mapping. **f**, **g** AC-HAADF-STEM images of Sn-Fe_2_O_3_-6. **h** Two profile lines were obtained from HAADF intensity analysis as labeled in **g**. **i** Atomic-resolution STEM-EDX elemental mapping for Fe, Sn in Sn-Fe_2_O_3_-6. **j** XRD patterns of Sn-Fe_2_O_3_-X. **k** Raman spectrum of Sn-Fe_2_O_3_-X

As illustrated in Fig. 1a, the atomically doped Sn sites in porous α-Fe_2_O_3_ were prepared through two steps, including the pre-substitution of Fe nodes with Sn atoms followed by oxidative annealing in air. During the crystallization process of Fe-based MOF (Fe-MIL-88B-NH_2_), Sn ions were in situ trapped by the organic ligands, which also served as the molecular fences to spatially separate those Sn atoms. Different molar ratios of Sn to Fe were prepared, and the obtained samples were denoted as Fe-MIL-88B-NH_2_-Sn-X (*X* = 0, 2, 4, 6, 8). After annealing in air, these materials transform into porous Fe_2_O_3_ with the immobilized Sn atoms (denoted as Sn-Fe_2_O_3_-X, *X* = 0, 2, 4, 6, 8). The X-ray diffraction (XRD) patterns of as-synthesized Fe-MIL-88B-NH_2_-Sn-X (Fig. S1) show that Fe-MIL-88B-NH_2_-Sn-0 matches well with the simulated results of the MIL-88B structure in the hexagonal space group of P6_3_/mmc [25]. Moreover, the MIL-88B structure remained almost unchanged after the introduction of Sn ions, suggesting that Sn ions did not significantly affect the crystallization process of Fe-MIL-88B-NH_2_ [26]. Nevertheless, compared to Fe-MIL-88B-NH_2_-Sn-0, the (101) diffraction peaks for Sn-doped MOF shift to a lower angle, and the shift degree is positively related to the addition of an amount of Sn ions. The gradually increased interplanar spacing of Fe-MIL-88B-NH_2_-Sn-X could be attributed to the successful substitution of Fe nodes by Sn atoms with a larger ionic radius [27]. The transmission electron microscopy (TEM) image in Fig. 1b shows that Fe-MIL-88B-NH_2_-Sn-0 has a spindle shape with average dimensions of 600 nm in length and 100 nm in width. From Figs. 1c and S2, it can be found that as the concentration of Sn ions increases, the morphology of the MOF changes from an elongated spindle shape to a stubby form. This transformation mainly originates from the altered nucleation behaviors of MOF crystals under the influence of Sn atoms [28].

Thermogravimetric analysis–derivative thermogravimetry (TGA-DTG) results (Fig. S3) confirm the complete transformation of MOF to MOS after annealing above 500 °C. The contents of Sn and Fe of Sn-Fe_2_O_3_-X were measured by inductively coupled plasma optical emission spectroscopy (ICP-OES) (Table S1), which are consistent with those in original Fe-MIL-88B-NH_2_-Sn-X samples. The TEM images of Sn-Fe_2_O_3_-X (Figs. 1d and S4) clearly show the formation of the porous entities composed of several nanoparticles, corresponding to the formation of porous MOS. Besides, the morphology of Fe_2_O_3_ and Sn-Fe_2_O_3_-X (*X* = 2, 4, 6, 8) did not significantly alter, suggesting the good compatibility of the synthesis strategy. The nitrogen adsorption–desorption isotherms of Sn-Fe_2_O_3_-X exhibit typical IV curves, indicating the presence of mesopores in the Sn-Fe_2_O_3_-X skeleton (Fig. S5a). The BET surface areas for Sn-Fe_2_O_3_-0, Sn-Fe_2_O_3_-2, Sn-Fe_2_O_3_-4, Sn-Fe_2_O_3_-6, and Sn-Fe_2_O_3_-8 were 50.7, 55.2, 61.5, 63.1, and 48.8 m^2^ g^−1^, respectively. Pore size distribution analysis revealed that all of the samples had a pore size of approximately 20 nm (Fig. S5b). The above results demonstrate that the MOF derivatives have a large specific area and extensive mesoporous structure, which facilitates the interactions between sensing material and gas molecules [29]. The selected area electron diffraction (SAED) pattern of Sn-Fe_2_O_3_-6 (inset in Fig. 1d) shows two distinct diffraction rings that could be indexed to the (104) and (110) crystalline planes of α-Fe_2_O_3_. These results further confirm the transformation of Fe-based MOF into α-Fe_2_O_3_ during the annealing process. Furthermore, the corresponding energy-dispersive X-ray (EDX) elemental mapping images (Fig. 1e) for Sn-Fe_2_O_3_-6 reveal the homogeneous distribution of Fe, O, and Sn elements throughout the entire porous entity. No large Sn nanoparticles or nanoclusters were observed. To further unravel the atomic distribution of Sn atoms in α-Fe_2_O_3_, atomic-resolution high-angle annular dark-field scanning transmission electron microscopy (HAADF-STEM) measurements were conducted. As illustrated in the HAADF-STEM images (Fig. 1f, g) and corresponding linear intensity profiles (Fig. 1h), the Sn atoms appear brighter than the surrounding Fe and O atoms due to their higher atomic number, which definitively identifies the atomical dispersion of Sn atoms within α-Fe_2_O_3_. This observation is supported by the merged atomic-resolution STEM-EDX elemental mapping images of Fe and Sn atoms (Figs. 1i and S6), which show that the majority of Sn atoms (cyan dots) are located at the positions belonging to Fe lattice atoms (purple dots), suggesting that the introduced Sn atoms mainly exist in the form of occupying Fe lattice sites.

XRD patterns of Sn-Fe_2_O_3_-X show characteristic diffraction peaks corresponding to α-Fe_2_O_3_ (PDF#33–0664, Fig. 1j). The diffraction peaks of Sn-Fe_2_O_3_-X present a gradual shift to a lower angle with the increase in Sn contents, which is in good agreement with the incorporation of Sn into the α-Fe_2_O_3_ lattice. Most importantly, no observable Sn crystal could be detected, which excludes the presence of Sn nanoparticles. Besides, a weak diffraction peak at 26° corresponding to the SnO_2_ (110) can be discerned for Sn-Fe_2_O_3_-8 sample, indicating that excessive Sn in MOF precursors would agglomerate and form Sn-related crystalline phase during the annealing process. Raman spectra of Sn-Fe_2_O_3_-X show two distinct vibrational modes of *A*_1g_ (221 and 485 cm^−1^) and *E*_g_ (288, 402, and 606 cm^−1^) (Fig. 1k), which are typical characters of hematite phase [30]. With the increase in Sn content, these peaks exhibit a slight redshift, indicating the weakening of the O–M–O bond. In addition, the Raman peak at 660 cm^−1^ originated from the disorder within the hematite crystal lattice and shows an increased intensity with the addition of Sn, again verifying the doping of Sn atoms into the α-Fe_2_O_3_ lattice [31, 32].

**Fig. 2:**
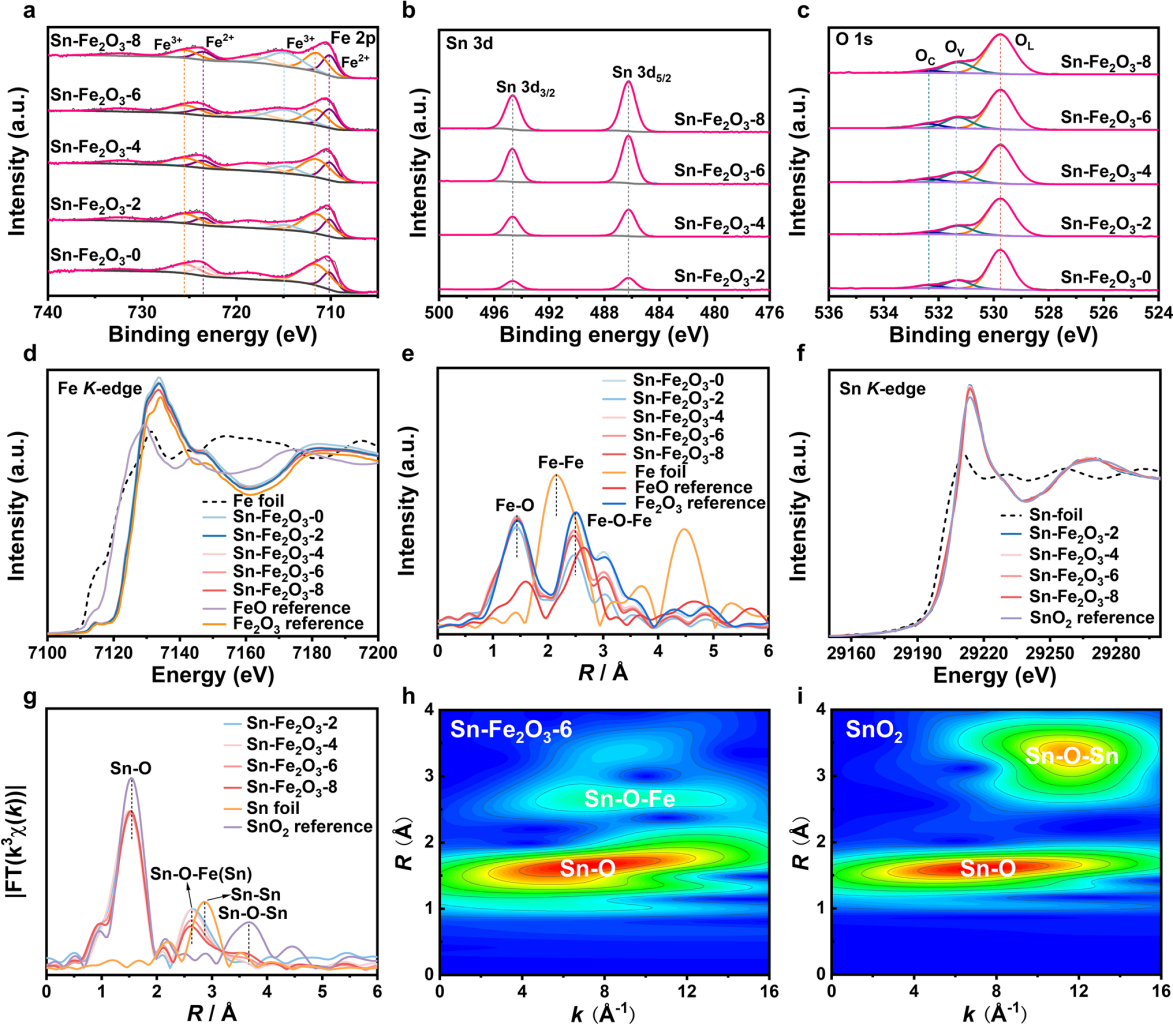
Chemical state and coordination structure of Sn-Fe_2_O_3_-X. High-resolution XPS spectra of **a** Fe 2*p*, **b** Sn 3*d*, and **c** O 1*s*. **d** Normalized Fe *K*-edge XANES spectra of Sn-Fe_2_O_3_-X, Fe foil, FeO, and Fe_2_O_3_ reference. **e** FT *k*^3^-weighted EXAFS spectra of Fe K-edge for Sn-Fe_2_O_3_-X, Fe foil, FeO, and Fe_2_O_3_. **f** Sn *K*-edge XANES of Sn-Fe_2_O_3_-X, Sn foil, and SnO_2_ reference. **g** FT *k*^3^-weighted EXAFS spectra of Sn K-edge for Sn-Fe_2_O_3_-X, Sn foil and SnO_2_ reference. Wavelet transform of the Sn *K*-edge for the EXAFS spectra of **h** Sn-Fe_2_O_3_-6 and **i** SnO_2_

X-ray photoelectron spectroscopy (XPS) measurements were carried out to investigate the elemental compositions and valence states of Sn-Fe_2_O_3_-X. The Fe 2*p* regions (Fig. 2a) can be fitted into two pairs of characteristic peaks, indicating the coexistence of Fe (II) (2*p*_3/2_ at 710.10 eV and 2*p*_1/2_ at 723.50 eV) and Fe (III) (2*p*_3/2_ at 711.5 eV and 2*p*_1/2_ at 725.42 eV). Notably, the ratio of Fe^2+^ is positively correlated with Sn contents. For example, the ratio of Fe^2+^ in Sn-Fe_2_O_3_-6 (45.1%) is much higher than that of Sn-Fe_2_O_3_-0 (27.3%) (Table S2), presumably due to the electron donor from the Sn atom to the neighboring Fe atom through the O bridge [33]. In high-resolution Sn 3*d* XPS spectra, two sets of doublet peaks were observed at 494.6 and 486.2 eV (Fig. 2b), which could be attributed to 3*d*_3/2_ and 3*d*_5/2_ peaks of Sn^4+^, respectively. Similarly, the asymmetric peaks of O 1*s* could be well fitted into three components (529.8, 531.3, and 532.4 eV), corresponding to the lattice oxygen (O_L_), oxygen vacancy (O_V_), and surface-chemisorbed oxygen (O_C_), respectively (Fig. 2c) [34]. The detailed fitting information for Fe 2*p* and O 1*s* XPS spectra is provided in Table S2. Notably, the ratio of O_V_ increases with the introduction of Sn, along with the increase in Fe^2+^ sites, which implies that atomically doping Sn significantly impacts the electronic structure of α-Fe_2_O_3_. The existence of Ov is also confirmed by the electron paramagnetic resonance (EPR) spectra (Fig. S7), where all Sn-Fe_2_O_3_-X samples display a symmetric signal at *g* = 2.005, attributed to the free electrons trapped in O_V_ [35]. Furthermore, the photoluminscence spectrum (PL) of Sn-Fe_2_O_3_-X is tested in Fig. S8. The two peaks at around 460 and 470 nm correspond to the emission of the oxygen vacancy [36]. With the incorporation of Sn atoms, the Sn-Fe_2_O_3_-X displayed lower PL intensity, demonstrating the suppressed electron–hole combination [37].

Synchrotron radiation-based X-ray absorption spectroscopy (XAS) technique, including X-ray absorption near edge structure (XANES) and extended X-ray absorption fine structure (EXAFS), was further exploited to probe the electronic structure and local coordination environment of Fe and Sn atoms in Sn-Fe_2_O_3_-X. The Fe K-edge XANES spectra and corresponding first derivatives are depicted in Figs. 2d and S9. The absorption edge energies (*E*_0_) are defined as the values of the first maxima in the first derivative spectra. In the case of Sn-Fe_2_O_3_-X, the *E*_0_ of Fe lies between that of FeO and Fe_2_O_3_, indicating the coexistence of + 2 and + 3 oxidation states of Fe in Sn-Fe_2_O_3_-X. Besides, the average oxidation state of Fe can be quantitatively estimated, which is based on the fact that the edge energy is directly proportional to the average oxidation state. Figure S9 shows that the average oxidation states of Fe in Sn-Fe_2_O_3_-X are 2.87, 2.86, 2.84, 2.86, and 2.83, respectively. The reason is likely attributed to the existence of oxygen vacancies and the change of the local electronic structure of Fe after Sn doping. The Fe K-edge Fourier transformed (FT) EXAFS spectra for Sn-Fe_2_O_3_-X (Fig. 2e) exhibit two main peaks at 1.44 and 2.48 Å, which can be attributed to Fe–O and Fe–O–Fe path, respectively. Compared to the Fe_2_O_3_ reference, the lower FT-EXAFS intensity of the Fe–O path for Sn-Fe_2_O_3_-X stems from the existence of O_V_, consistent with their smaller coordination numbers of Fe–O path determined by EXAFS fitting (Fig. S11 and Table S3). Additionally, the absorption edges of Sn K-edge XANES spectra (Fig. 2f) and first derivative of Sn K-edge XANES (Fig. S10) for Sn-Fe_2_O_3_-X share a close energy position with that of SnO_2_ reference, suggesting the Sn valences in Sn-Fe_2_O_3_-X were mainly + 4. The Sn EXAFS fitting results (Fig. S12 and Table S4) showed that Sn atoms with a similar coordination environment to Fe atoms remained stable. From corresponding FT-EXAFS spectra (Fig. 2g), a small peak at 2.65 Å can be discerned, which is not detected in Sn foil and SnO_2_ reference but shows a similar radical distance with that of Fe–O–Fe path in α-Fe_2_O_3_. Furthermore, this unique path can also be observed from the Wavelet transform EXAFS (WT-EXAFS) in Fig. 2h, i. Clearly, the WT-EXAFS plot of the SnO_2_ reference shows two distinct contour maximums, which can be ascribed to the contributions of Sn–O and Sn–O–Sn. In contrast, except for the contour maximum from Sn–O path, the WT-EXAFS plot of Sn-Fe_2_O_3_-6 shows a unique maximum that is located at a lower R- and k-position than those of Sn–O–Sn path, implying that this path originates from the scattering path between Sn atom and low-Z Fe atom. These findings demonstrate the formation of Sn–O–Fe sites, which match well with the identified doping sites of Sn atom in the α-Fe_2_O_3_ lattice.

### Sensing Performance of Sn-Fe_2_O_3_-X

**Fig. 3:**
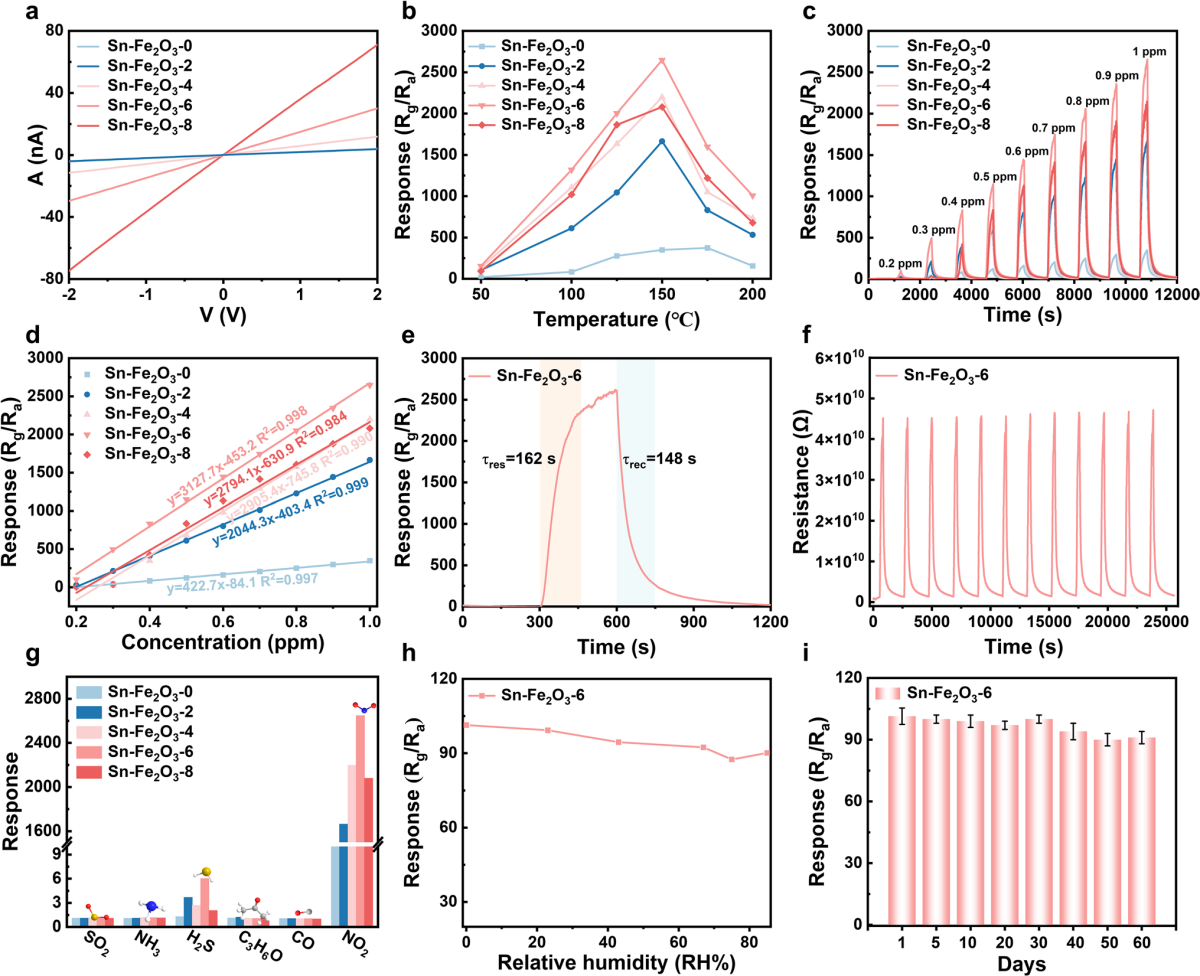
Chemiresistive sensing performance of Sn-Fe_2_O_3_-X. **a** *I–V* curves of Sn-Fe_2_O_3_-X at 150 °C. **b** Response of Sn-Fe_2_O_3_-X to 1 ppm NO_2_ at different working temperatures. **c** Dynamic response curves of Sn-Fe_2_O_3_-X to 0.2–1 ppm NO_2_ at 150 °C. **d** Linear dependence of concentration on response. **e** Response–recover curves of Sn-Fe_2_O_3_-X to 1 ppm NO_2_ at 150 °C. **f** Cycle reproducibility of Sn-Fe_2_O_3_-6 to 0.2 ppm NO_2_ at 150 °C. **g** Response of Sn-Fe_2_O_3_-X to different gases at 150 °C. **h** The sensitivity of Sn-Fe_2_O_3_-6 to 0.2 ppm NO_2_ in different humidity conditions **i** Long-term stability of Sn-Fe_2_O_3_-6 toward 0.2 ppm NO_2_

The contribution of atomically doping Sn sites in porous α-Fe_2_O_3_ sensing layer to gas sensing performance was experimentally evaluated by a series of chemiresistive sensing measurements using NO_2_ as the target gas. The sensing materials were first drop-coated on prefabricated alumina substrates with interdigitated electrodes. After aging treatment, the current–voltage (I–V) curves of these devices were collected. As shown in Fig. S13, we have tested the Sn-Fe_2_O_3_-X based gas sensor under different temperatures to study the semiconducting properties. All samples displayed negative temperature coefficient effect, which is consistent with the characteristic of semiconductor [38]. The *I–V* curves presented in Fig. 3a show a typical symmetrical line profile, indicative of the good ohmic contact between the sensing materials layer and electrodes [39]. Generally, the sensing performance of semiconductor-based gas sensors shows an intimate correlation with the operating temperature, owing to its influence on the carrier concentration of semiconductors and the reactivity of gas molecules with the sensing materials. To identify the optimal working temperature, the Sn-Fe_2_O_3_-X was exposed to 1 ppm of NO_2_ gas in a wide temperature range (50–200 °C). As shown in Fig. 3b, the optimal working temperature for Sn-Fe_2_O_3_-0 is 175 °C, while a lower optimal working temperature (150 °C) is recognized for Sn-Fe_2_O_3_-2, 4, 6, 8. The decrease in optimal working temperature can be attributed to the reduced activation energy of NO_2_ molecules on Sn-contained sensing materials.

The dynamic response/recovery curves of the Sn-Fe_2_O_3_-X in response to NO_2_ concentrations from 0.2 to 1 ppm are shown in Figs. 3c and S14. The Sn-Fe_2_O_3_-X exhibited a typical n-type response to NO_2_ gas and responses of all samples increase with the increasing concentrations of NO_2_ gas. For 1 ppm NO_2_, the Sn-Fe_2_O_3_-0 (pure α-Fe_2_O_3_) shows a superior response value to conventional MOS-based sensors, presumably due to their structural advantages (e.g., mesopores and O_V_). After the introduction of Sn, the response values are found to be dramatically improved, signifying the essential role of atomically dispersed Sn sites in the sensing process. Among them, Sn-Fe_2_O_3_-6 exhibits the highest response (2646.6) to 1 ppm NO_2_, far surpassing Sn-Fe_2_O_3_-0 (349.9), Sn-Fe_2_O_3_-2 (1664.0), Sn-Fe_2_O_3_-4 (2197.3), and Sn-Fe_2_O_3_-8 (2147.6), denoting the optimum doping ratio of Sn for gas sensing is 6 at%. In addition, the response values of all samples display good linearity concerning NO_2_ concentration (Fig. 3d), which allows for the quantitative estimation of NO_2_ levels in the environment. To evaluate the response and recovery speed of the sensing materials, response/recovery time (*τ*_res_/*τ*_recov_) was measured at 1 ppm NO_2_, as shown in Figs. 3e and S16. The *τ*_res_/*τ*_recov_ values of Sn-Fe_2_O_3_-X are summarized in Table S5. Clearly, the introduction of Sn sites significantly reduces their response and recovery time to 1 ppm NO_2_. The active sites (Sn–O–Fe) provide more gas adsorption and reaction sites, thereby accelerating the response process. The shortened recovery time after single-atom doping is likely due to the reduced energy barrier for NO_2_ desorption at the Sn–O–Fe active sites. The presence of single Sn atoms promotes the efficient release of NO_2_ molecules from the surface, thereby accelerating the recovery process. It is widely acknowledged that the practical application of NO_2_ sensors is typically limited by their prolonged recovery time due to the strong oxidizing properties of the NO_2_ molecule, whereas the atomically dispersed Sn sites in α-Fe_2_O_3_ can not only enhance the sensitivity to NO_2_ but also reduce its recovery time.

The repeatability of Sn-Fe_2_O_3_-6 was evaluated using 0.2 ppm NO_2_. As displayed in Fig. 3f, the resistance of the fabricated device remained constant, showing reversible and stable characteristics during each cyclic exposure. The selectivity of Sn-Fe_2_O_3_-X was also investigated by exposing it to 0.2 ppm NO_2_ and 1 ppm of other gases (SO_2_, NH_3_, H_2_S, C_3_H_6_O, CO) at 150 °C. Figure 3g shows that the Sn-Fe_2_O_3_ samples exhibited superior selectivity for NO_2_, demonstrating their excellent anti-interference ability. To further examine the influence of humidity on the sensitivity of Sn-Fe_2_O_3_-6, the response of the Sn-Fe_2_O_3_-6 toward 0.2 ppm NO_2_ was tested under different humidity conditions. As shown in Fig. 3h, the response remains constant as humidity increases, indicating that the Sn-Fe_2_O_3_-6 exhibits excellent moisture resistance. Furthermore, the long-term stability of the Sn-Fe_2_O_3_-6 was tested under 0.2 ppm NO_2_ at 150 °C, showing no significant deterioration in response over two months (Fig. 3i), further confirming its durability.

**Fig. 4:**
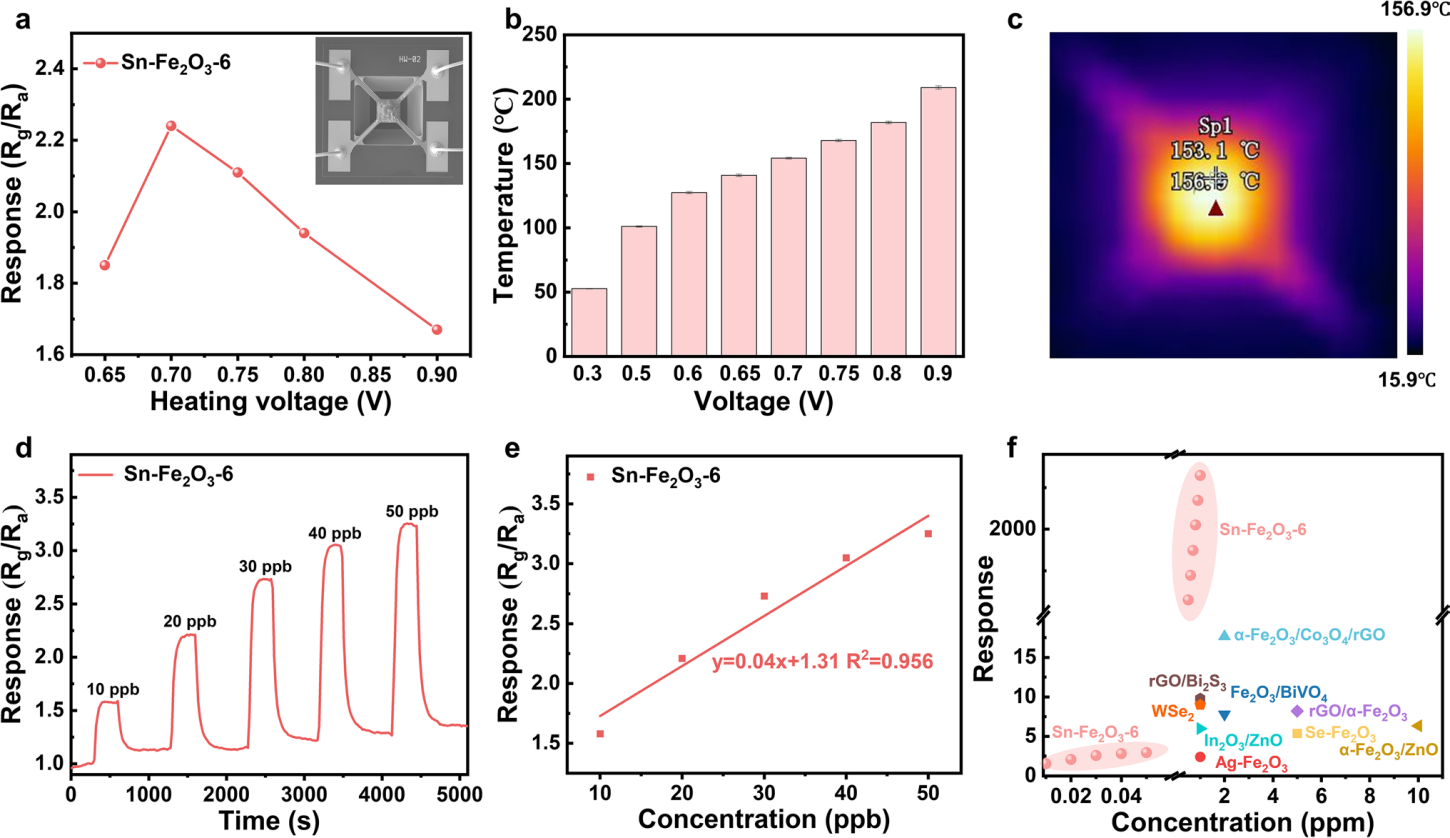
Sensing performance of Sn-Fe_2_O_3_-6-based MEMS gas sensor. **a** The response of the Sn-Fe_2_O_3_-6-based MEMS gas sensor to 20 ppb NO_2_ under different heating voltages along with the SEM image (inset) of sensing layer on the MEMS microheater. **b** The actual acquired temperatures under different heating voltages. **c** Infrared thermal map over the sensing area under 0.70 V. **d** Dynamic sensing characteristics of the Sn-Fe_2_O_3_-6-based MEMS sensor toward 10–50 ppb NO_2_. **e** Correlation curve of the response of the Sn-Fe_2_O_3_-6-based MEMS sensor with NO_2_ concentration. **f** Comparison of gas sensing performances of NO_2_ sensing materials reported in recent literature and in this work

For potential commercial applications, the microelectromechanical system (MEMS)-based gas sensor was fabricated by using Sn-Fe_2_O_3_-6 as sensing material. The MEMS gas sensors possess various advantages, such as compact size, low power consumption, and easy integration. The structure schematic diagram of the MEMS hotplate is shown in Fig. S17a, and the hotplates were fabricated via the MEMS process, as described in Supporting Information. The sensing material was firstly coated on the testing electrodes (100 × 100 μm^2^ in sensing area) of MEMS hotplate via an electrohydrodynamic (EHD)-based dispensing coating route by a dispensing machine. The hotplate with the sensing materials was packed in a ceramic cartridge to form a gas sensor (3 × 3 × 1.3 mm^3^ in L × W × H) as shown in Fig. S17b. Figure 4a displays the response of the Sn-Fe_2_O_3_-6-based MEMS sensors to 20 ppb NO_2_ under different heating voltages, in which the actual temperatures corresponding to different heating voltages were measured using an infrared thermometer (Figs. 4b and S18). The Sn-Fe_2_O_3_-6-based MEMS sensor exhibits the highest response value (2.24) at 0.7 V, corresponding to 153 °C in temperature (Fig. 4c**)**, which is consistent with the optima working temperature of the corresponding sensing material on alumina substrate. The power consumption of the Sn-Fe_2_O_3_-6-based MEMS gas sensor was estimated at only 8.4 mW, which is much lower than those of commercially available MOS-based MEMS gas sensors (around 40 mW). The dynamic response/recovery curves of Sn-Fe_2_O_3_-6-based MEMS sensor toward ultra-low concentration of NO_2_ (10–50 ppb) are shown in Fig. 4d. Obviously, the MEMS sensor exhibits an ideal response (1.58) toward 10 ppb NO_2_ and displays a good linear relationship between response value and gas concentrations (Fig. 4e). These results demonstrate the potential commercialization of Sn-Fe_2_O_3_-6 for detecting trace amounts of NO_2_ using advanced MEMS-based sensor platforms. Overall, as shown in Fig. 4f and Table S6, the MOF-derived oxygen vacancy-rich α-Fe_2_O_3_ with atomically dispersed Sn sites in this work exhibits superior sensitivity to NO_2_ compared to most of the previously reported materials [40–48]. In addition to this, it also can test ultra-low concentration of NO_2_ gas (10 ppb) instead of the theoretically calculated limit of detection.

### Sensing Mechanism of Sn-Fe_2_O_3_-X

**Fig. 5:**
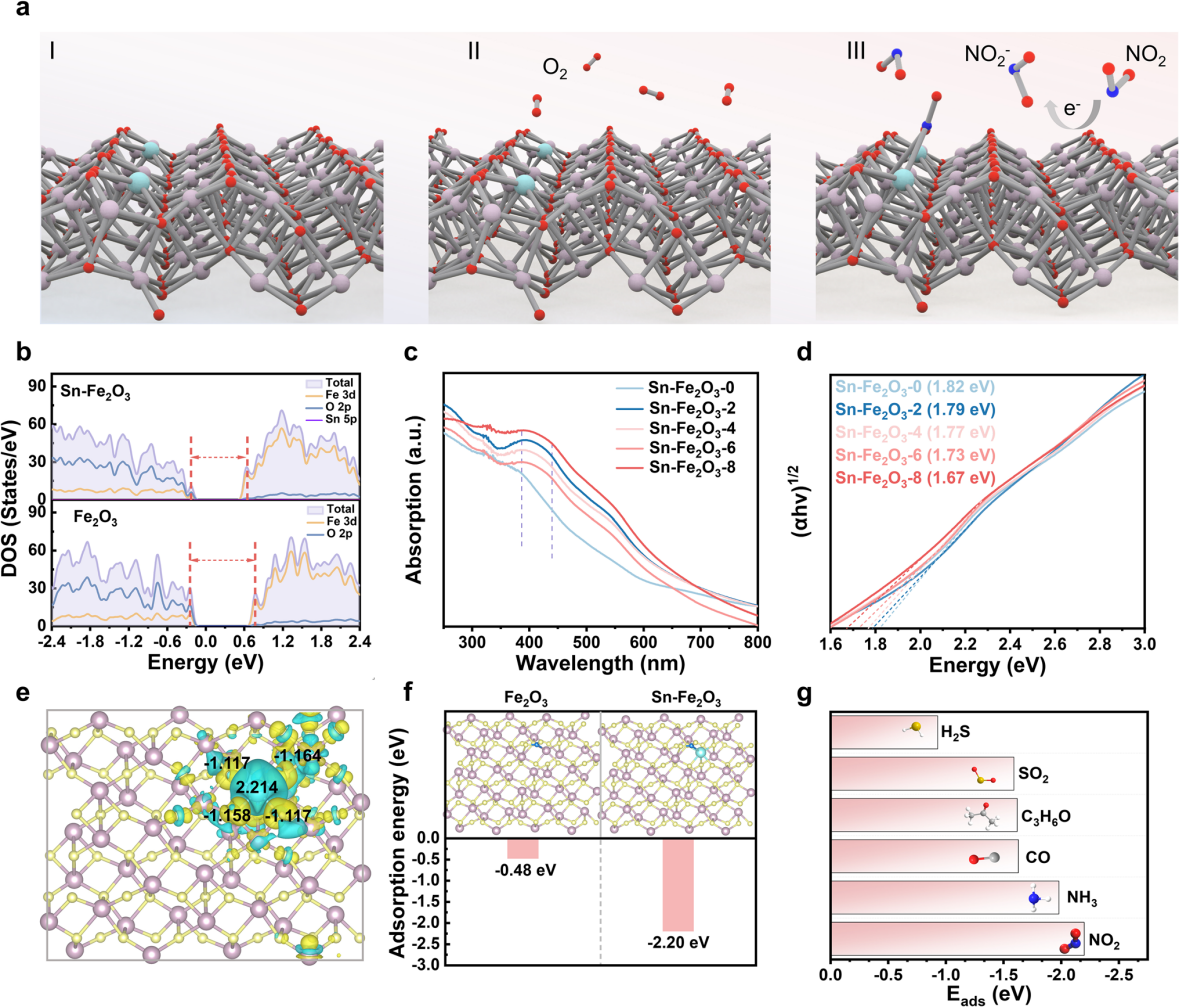
Investigation of the role of Sn-single sites in sensing progress. a Sensing process diagram of Sn-Fe_2_O_3_ for NO_2_. **b** Density of state of Fe_2_O_3_ and Sn-Fe_2_O_3_ obtained from DFT calculations. **c** UV–vis absorption spectra of Sn-Fe_2_O_3_-X. **d** Tauc plot according to the UV–vis absorption spectra to estimate the bandgap. **e** Charge density of Sn-Fe_2_O_3_ and net charge of Sn and surrounding O atoms. Charge accumulation and depletion are indicated by yellow and cyan areas, respectively. **f** Adsorption energies of NO_2_ on the optimum structures of Fe_2_O_3_ and Sn-Fe_2_O_3_. **g** Adsorption energies of different gases on the optimum structure of Sn-Fe_2_O_3_

In metal oxide semiconductor-based gas sensors, the sensing mechanism primarily relies on changes in resistance after interaction with target gas molecules [49]. Figure 5a depicts a schematic of the gas sensing process of Sn-Fe_2_O_3_-X toward NO_2_. Firstly, the oxygen molecules (O_2_) from air atmosphere adsorb on Sn-doped Fe_2_O_3_, forming adsorbed oxygen species (${{\text{O}}_{2}}^{-}$) [50]. The porous Fe_2_O_3_ with rich Ov is expected to provide abundant adsorbed oxygen species [51, 52]. As a strong oxidative electron acceptor, NO_2_ has a stronger electron affinity. When exposed to NO_2_ gas, these adsorbed oxygen species (mainly ${{{\text{O}}_{2}}^{-}}_{\text{(ads)}}$) interact with NO_2_, or the NO_2_ molecule directly adsorbs onto the sensing materials, extracting electrons from the Sn-doped Fe_2_O_3_ conduction band to form ${{{\text{NO}}_{2}}^{-}}_{\text{(ads)}}$ and leading to an increase in resistance, which can be measured by the external circuit of corresponding gas sensors. To investigate the effect of dispersed Sn SAs, the total density of states (DOS) of α-Fe_2_O_3_ before and after Sn doping were calculated (Fig. 5b). Clearly, the energy difference between the top of the valence band (VB) and the bottom of the conduction band (CB) for Sn-Fe_2_O_3_ is smaller than that of pure Fe_2_O_3_, mainly due to the disturbed charge balance caused by Sn doping. The DOS demonstrated that the introduction of single Sn atoms into the Fe_2_O_3_ lattice resulted in a narrowing of the bandgap. The decreased bandgap of sensing materials was also verified by the UV–vis spectra and corresponding Tauc plots, as shown in Fig. 5c, d. Therefore, the introduction of Sn atoms into Fe_2_O_3_ has been demonstrated to affect the local electronic structure, consequently reducing the bandgap. The reduction in the bandgap of Fe_2_O_3_ after Sn doping facilitates improved charge carrier mobility and enhanced surface reactivity, which collectively contribute to the superior NO_2_ sensing performance at low operating temperatures. Based on the aforementioned sensing process, it can be known that the adsorption of gas molecules on sensing materials is significant to the apparent sensing performance. Furthermore, density functional theory (DFT) calculations were performed to understand the performance enhancement mechanisms introduced by atomically dispersed Sn sites. As shown in Fig. S19, two slab models, Fe_2_O_3_(110) and Fe_2_O_3_(110)-Sn ((Fe_2_O_3_(110) with one Fe atom replaced by Sn atom), were constructed. The charge density difference was first calculated to elucidate the charge transfer between Sn atom and Fe_2_O_3_. The yellow and cyan lobes in Fig. 5e represent the charge accumulation and depletion after the introduction of Sn atom. Bader charge analysis shows the net charge of Sn atom (2.22e) and surrounding O atoms (− 1.139e), implying the obvious charge transfer from Sn to the surrounding Fe_2_O_3_ lattice. The electrons migrate from the Sn atom to the neighboring O atoms, creating a positively charged center that enhances NO_2_ adsorption. This result can explain the appearance of Sn^4+^, as detected by XPS and XANES. The adsorption energies (*E*_ads_) were further calculated to evaluate the adsorption behaviors of NO_2_ on Fe_2_O_3_ and Sn-doped Fe_2_O_3_. As shown in Fig. 5f, Sn and Fe atoms in Sn–O–Fe sites participate in the adsorption of NO_2_ (N on Sn atom, O on Fe atom), while two Fe atoms of Fe_2_O_3_ function as the adsorption sites. The calculated NO_2_ adsorption energy for Sn-Fe_2_O_3_ is − 2.20 eV, which is more negative than that for pure Fe_2_O_3_ (− 0.48 eV). It is suggested that the atomically dispersed Sn atom provides more favorable adsorption sites for NO_2_ molecules, which contributes to the high response value toward NO_2_. Besides, the adsorption behaviors of other gases on Sn-doped Fe_2_O_3_ were also investigated, as displayed in Figs. 5g, S20, and Table S7. The adsorption energies for other gases are much more positive than that for NO_2_, indicating their lower adsorption intensities on Sn-doped Fe_2_O_3_. Overall, the atomically dispersed Sn atom within Sn-doped Fe_2_O_3_ plays a critical role in improving the sensitivity and selectivity of corresponding gas sensors toward NO_2_, which is mainly realized by providing both favorable adsorption sites and available conductive electrons.

## Conclusions

In this work, atomically dispersed Sn sites within porous α-Fe_2_O_3_ were successfully synthesized through the in situ trapping of Sn atoms during MOF crystallization and subsequent atomic dispersion during the annealing process. Comprehensive structural evidence from STEM and XAS have clearly demonstrated that Sn atoms were incorporated into the porous Fe_2_O_3_ matrix as atomically dispersed sites, forming Sn–O–Fe configurations. The Sn-Fe_2_O_3_-6-based MEMS gas sensor exhibits exceptional sensing performance toward NO_2_ gas at a low operating temperature of 150 °C, achieving a high response (2646.6–1 ppm NO_2_), an ultra-low detection limit (10 ppb), excellent selectivity, and long-term stability. DFT calculations revealed that the enhanced sensing performance of Sn-doped Fe_2_O_3_ is attributed to the specific adsorption sites and additional conductive electrons provided by atomically dispersed Sn atoms. These findings highlight the potential of atomically dispersed sites for high-performance gas sensors and offer a strategic pathway for designing advanced sensing materials for practical applications.

## Supplementary Information

Below is the link to the electronic supplementary material.

Supplementary file1 (DOCX 13038 KB)
