# Supplementary material for: Specific Sn–O–Fe Active Sites from Atomically Sn-Doping Porous Fe2O3 for Ultrasensitive NO2 Detection
Source: Nanomicro Lett. 2025 May 26;17:276. doi: 10.1007/s40820-025-01770-9 (PMC12104129; doi:10.1007/s40820-025-01770-9)
Supplement: Supplementary file 1 — Supplementary file1 (DOCX 13038 KB) [file 40820_2025_1770_MOESM1_ESM.docx]

Supporting Information for

**Specific Sn-O-Fe Active Sites from Atomically-Sn-Doping Porous Fe_2_O_3_ for Ultrasensitive NO_2_ Detection**

Yihong Zhong^1^, Guotao Yuan^2^, Dequan Bao^3^, Yi Tao^1^, Zhenqiu Gao^1^, Wei Zhao^1^, Shuo Li^1^, Yuting Yang^1^, Pingping Zhang^4^, Hao Zhang^1^*, Xuhui Sun^1^*

^1^Institute of Functional Nano and Soft Materials (FUNSOM), Jiangsu Key Laboratory for Carbon-Based Functional Materials and Devices, Jiangsu Key Laboratory of Advanced Negative Carbon Technologies, Soochow University, Suzhou 215123, P. R. China

^2^College of Chemistry and Environmental Engineering, Shenzhen University, Shenzhen 518060, P. R. China

^3^The Key Laboratory of Rare Earth Functional Materials and Applications, Zhoukou Normal University, Zhoukou 466000, P. R. China

^4^Suzhou Huiwen Nanotechnology Co., Ltd. Suzhou 215000, P. R. China

* Corresponding authors. E-mail: [haozhang@suda.edu.cn](mailto:haozhang@suda.edu.cn) (Hao Zhang); [xhsun@suda.edu.cn](mailto:xhsun@suda.edu.cn) (Xuhui Sun)

**S1** **Experimental Section**

***Characterization:*** The actual molar ratio of Sn to Fe atoms in as-prepared Sn-Fe_2_O_3_-X was determined by inductivity coupled plasma optical emission spectroscopy (ICP-OES, PerkinElmer Avio 200). The morphology and structure of samples were measured by high-resolution transmission electron microscope (HRTEM) (FEI Talos F200X and Thermo Fisher Scientific Spectra 300S/TEM). Thermogravimetric analysis (TGA) was investigated through a TGA1 (Mettler Toledo) apparatus. N_2_ sorption isotherms under 77 K were tested (Micromeritics ASAP 2050 Xtended Pressure Sorption Analyzer). The crystal structure and phase were determined by powder X-ray diffraction (PANalytical X-ray diffractometer with Cu Kα radiation, λ=0.154 nm) and Raman spectrum (JY HR800). X-ray photoelectron spectroscopy (XPS) was measured using a KRATOS AXIS Ultra DLD X-ray electronics photoelectron spectrometer using a monochromatic Al Kα (1486.6 eV) X-ray source with a power of 96 W (12 kV and 8 mA), where binding energies were calibrated by referencing the adventitious C 1s peak (Bes=284.8 eV). The X-ray absorption spectra (XAS) of Fe and Sn K-edge were carried out at the Shanghai Synchrotron Facilities (SSRF, BL14W and BL20U1). The existence of oxygen vacancy was investigated by EPR spectroscopy at room temperature (JEOL, JES-X320). The band structure of the semiconductor metal oxides was studied by the UV-Vis absorption spectrum performed using the UV-Vis-NIR Spectrophotometer (PE750). The photoluminescence (PL) of Sn-Fe2O3-X was tested with FL-TCSPC (HORIBA Jobin Yvon).

***Gas Sensing Performance Measurements:*** The sample was mixed with deionized water into a slurry and then the result slurry was coated on the alumina substrates (13.4 mm × 7 mm) with a pair of interdigital Ag-Pd electrodes to form a uniform film, which was subsequently dried at 80 °C to obtain the sensor. Before testing the sensing performance, the gas sensor was aged in an oven at 200 °C for 3 days. A CGS-MT Mini Multifunctional Probe Station (Beijing Tech Co., Ltd., China) was used to measure the sensing performance of α-Fe_2_O_3_ and Sn-Fe_2_O_3_. The test was performed using a dynamic test method: the concentration of testing gases was acquired by diluting the standard gas with dry air and gas flows were accurately controlled through a mass flow controller (MFC). Herein, for quantitative analysis of the gas sensors, the response of the sensor was defined as R_g_/R_a_ for oxidizing gas (NO_2_) or R_a_/R_g_ for reducing gas (H_2_S, acetone, NH_3_, CO, SO_2_), where R_a_ and R_g_ denoted the resistance of the sensor in dry air and the target reducing/oxidizing gas, respectively. In addition, the response/recovery time is defined as the time required to achieve a 90% variation in the sensor resistance value after the target gas is carried in/out.

***MEMS Gas Sensor Fabrication:*** Micro-hotplate structures are fabricated via surface silicon micro-machining, resulting in a suspended platform with an embedded heater and thermally isolated from the substrate. The heating electrodes and the interdigital sensing electrodes were deposited with the same metal deposition step. Then, SiNx layer was deposited to insulate the heating electrodes and sensing electrodes. The sensing materials and Triton X-100 were grounded to form a uniform paste, and the paste was coated on the surface of the interdigital electrodes. The device was then aged in a muffle furnace to improve its stability. Finally, the MEMS-based gas sensor was fabricated by connecting the MEMS device with the external circuit through wire bonding. The sensing performance of MEMS gas sensor was tested by the static system consisting of a power supply, test chamber, Keithley 2700, and a computer. The gas was injected into chamber through the syringe and its concentration can be determined based on the ratio of injected gas volume to chamber volume.

***Computational details:*** In Density Functional Theory (DFT) calculations, we constructed a slab model of Fe_2_O_3_ (110) 1×2×1 supercell based on α-Fe_2_O_3_ hexagonal bulk structure. One Sn atom was introduced to replace the Fe atom to construct a slab model of Fe_2_O_3_ (110)-Sn. Structural optimizations were performed by the Vienna Ab-initio Simulation Package (VASP) with the projector augmented wave (PAW) method. The exchange-functional was treated using the Perdew-Burke-Ernzerhof (PBE) functional, in combination with the DFT-D3 correction. The cut-off energy of the plane-wave basis was set at 450 eV in structural optimization. For the optimization of the lattice size of the α-Fe_2_O_3_ bulk structure, the Brillouin zone integration was performed with a Monkhorst-Pack k-point mesh of 9×9×2. For the optimization of the geometry of slab models, the Brillouin zone integration was performed with a Monkhorst-Pack k-point mesh of 2×1×1. The self-consistent calculations applied a convergence energy threshold of 10^-5^ eV. The equilibrium geometries and lattice constants were optimized with maximum stress on each atom within 0.02 eV Å^-1^. Spin polarization was adopted to describe the antiferromagnetism of Fe_2_O_3_(110). Specifically, magnetic moment was set at 2 on half of Fe atoms, and -2 on the other half of Fe atoms. Hubbard U correction was added to describe strong interactions of Fe-3d orbitals, where U_Fe_ = 4.0eV. The density of the state of slab models was obtained by vaspkit interface. Isosurface level of charge density difference of Fe_2_O_3_ (110)-Sn was set at 0.02 e Å^-3^. The adsorption energies ($\text{E}_{\text{ads}}$) would be defined as follows:

$$\text{E}_{\text{ads}}\text{=}\text{E}_{\text{(s-m)}}\text{-}\text{E}_{\text{S}}\text{-}\text{E}_{\text{m}}$$

Where $\text{E}_{\text{s-m}}$represents the total energy of the slab model of Fe_2_O_3_(110) and Fe_2_O_3_(110)-Sn adsorbing NO_2_, $\text{E}_{\text{s}}$ represents the total energy of the slab model of Fe_2_O_3_(110) and Fe_2_O_3_(110)-Sn, $\text{E}_{\text{m}}$represents the total energy of the NO_2_ molecule in its gas phase. The configuration of NO_2_ was optimized in a 20×20×20 Å^3^ box.

**S2 Supplementary Figures and Tables**


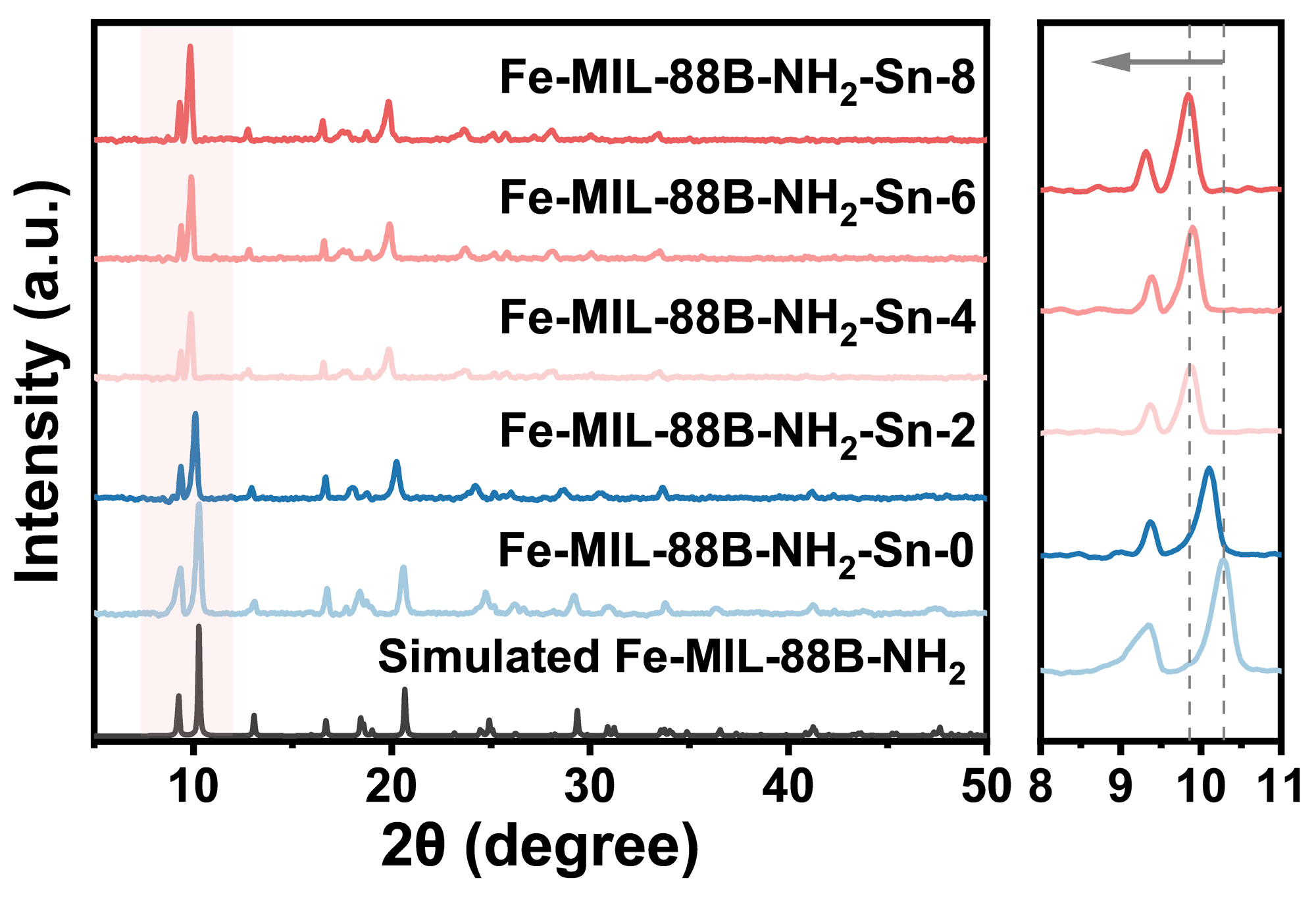


**Fig. S1** XRD patterns of Fe-MIL-88B-NH_2_-Sn-X


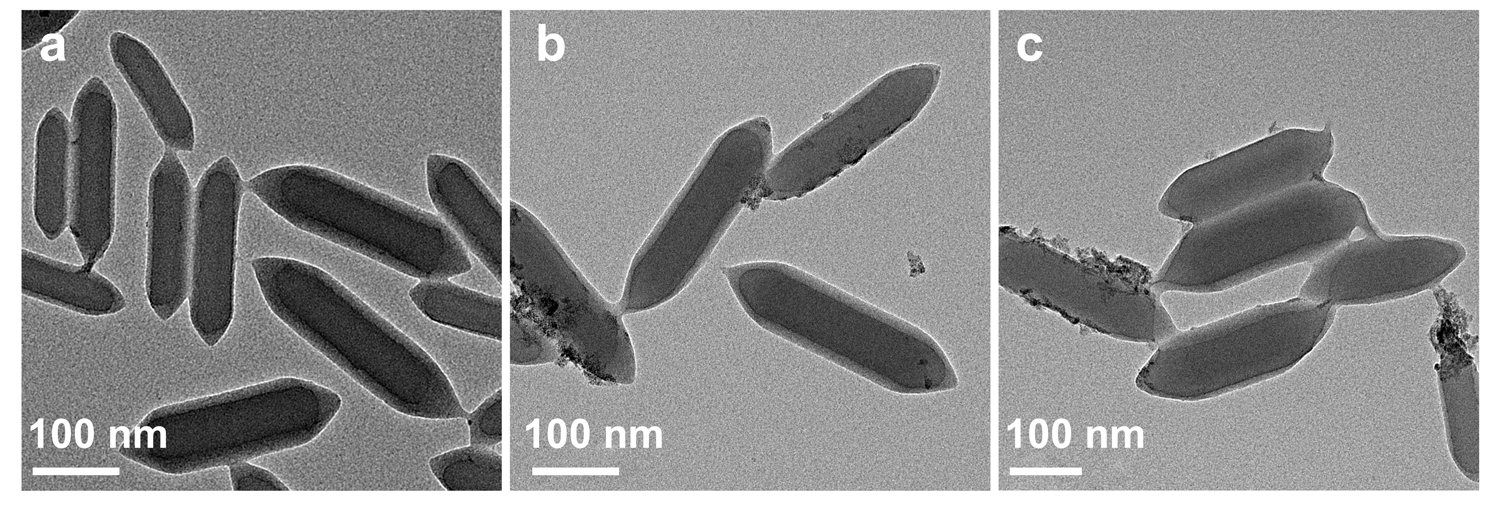


**Fig. S2** TEM images of **a** Fe-MIL-88B-NH_2_-Sn-2, **b** Fe-MIL-88B-NH_2_-Sn-4, and **c** Fe-MIL-88B-NH_2_-Sn-8

**
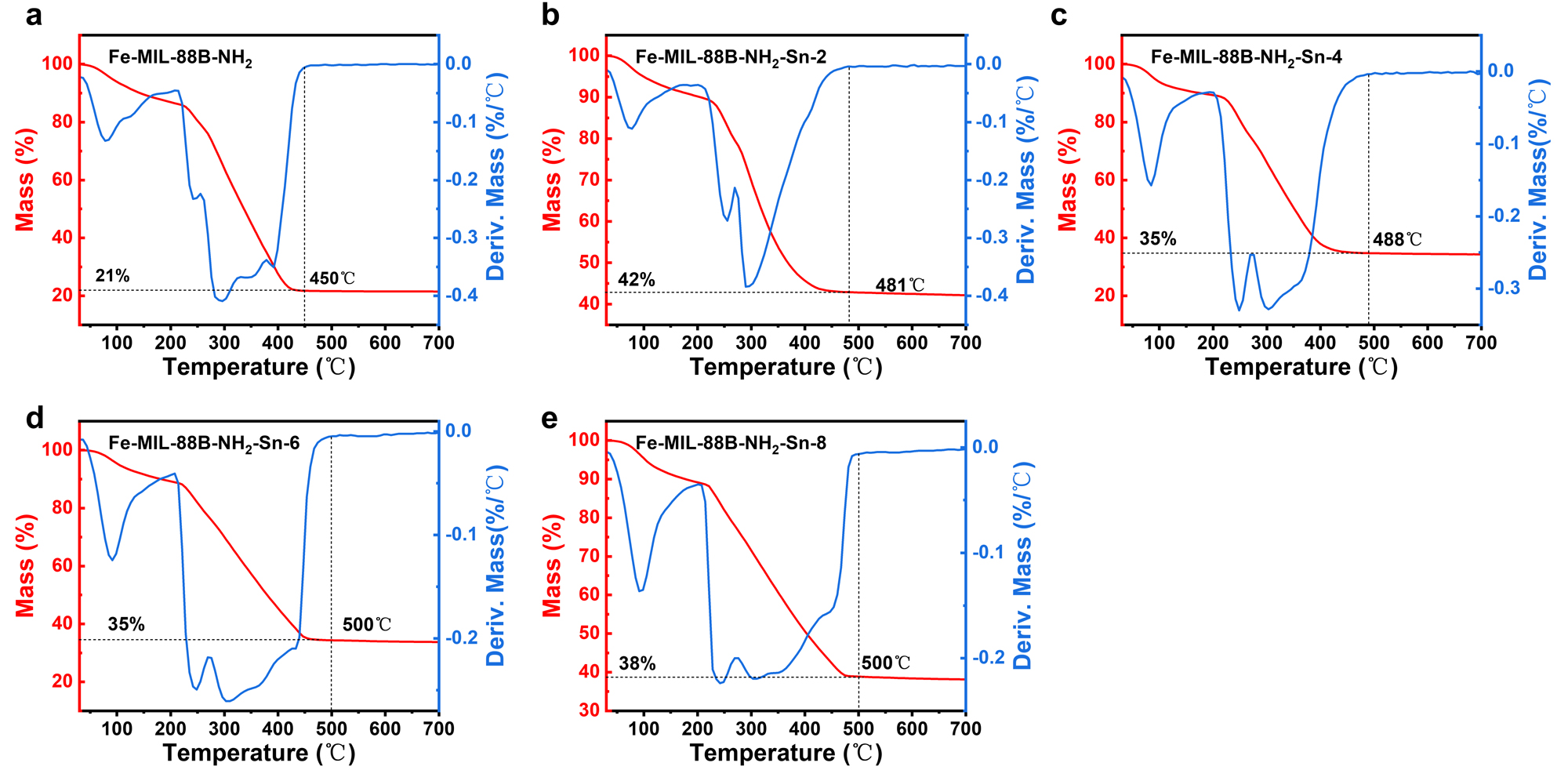
**

**Fig. S3** TGA-DTG of **a** Fe-MIL-88B-NH_2_, **b** Fe-MIL-88B-NH_2_-Sn-2, **c** Fe-MIL-88B-NH_2_-Sn-4, **d** Fe-MIL-88B-NH_2_-Sn-6, **e** Fe-MIL-88B-NH_2_-Sn-8.


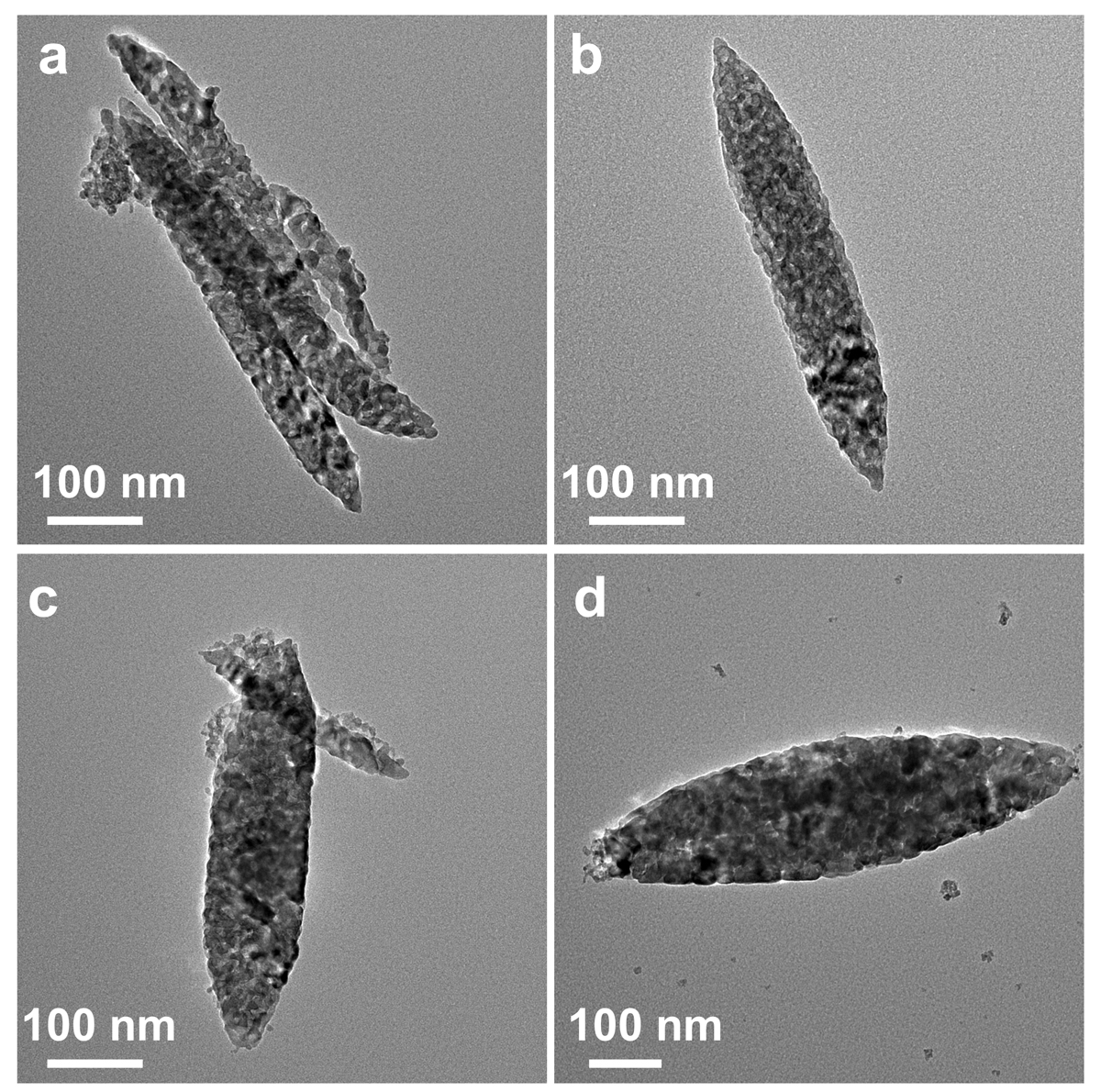


**Fig. S4** TEM images of **a** Sn-Fe_2_O_3_-0, **b** Sn-Fe_2_O_3_-2, **c** Sn-Fe_2_O_3_-4, **d** Sn-Fe_2_O_3_-8


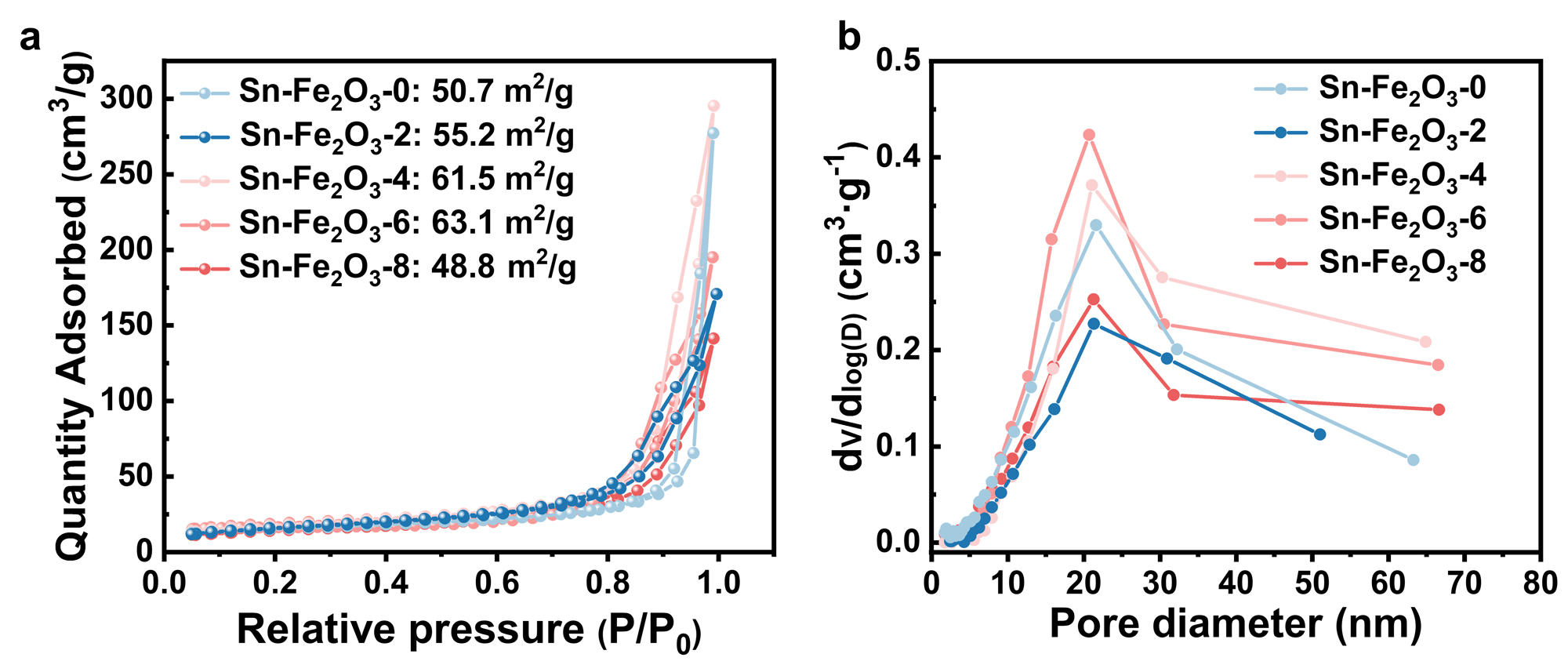


**Fig. S5 a** N_2_ adsorption-desorption isotherms and corresponding, **b** BJH adsorption pore size distributions of Sn-Fe_2_O_3_-0, Sn-Fe_2_O_3_-2, Sn-Fe_2_O_3_-4, Sn-Fe_2_O_3_-6, and Sn-Fe_2_O_3_-8


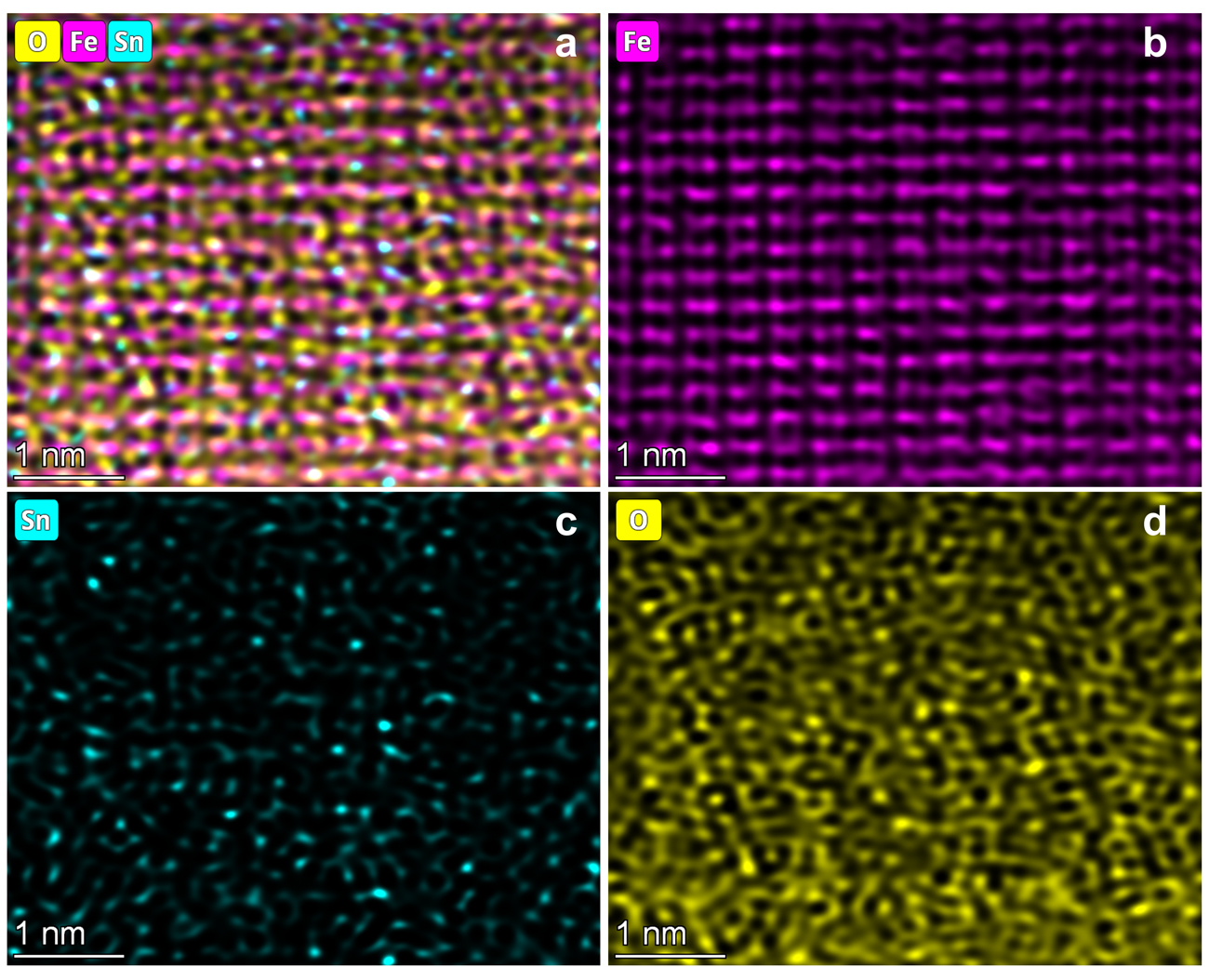


**Fig. S6** Atomic-resolution HAADF-EDX elemental mappings of **a** mixing, **b** Fe, **c** Sn, **d** O in Sn-Fe_2_O_3_-6


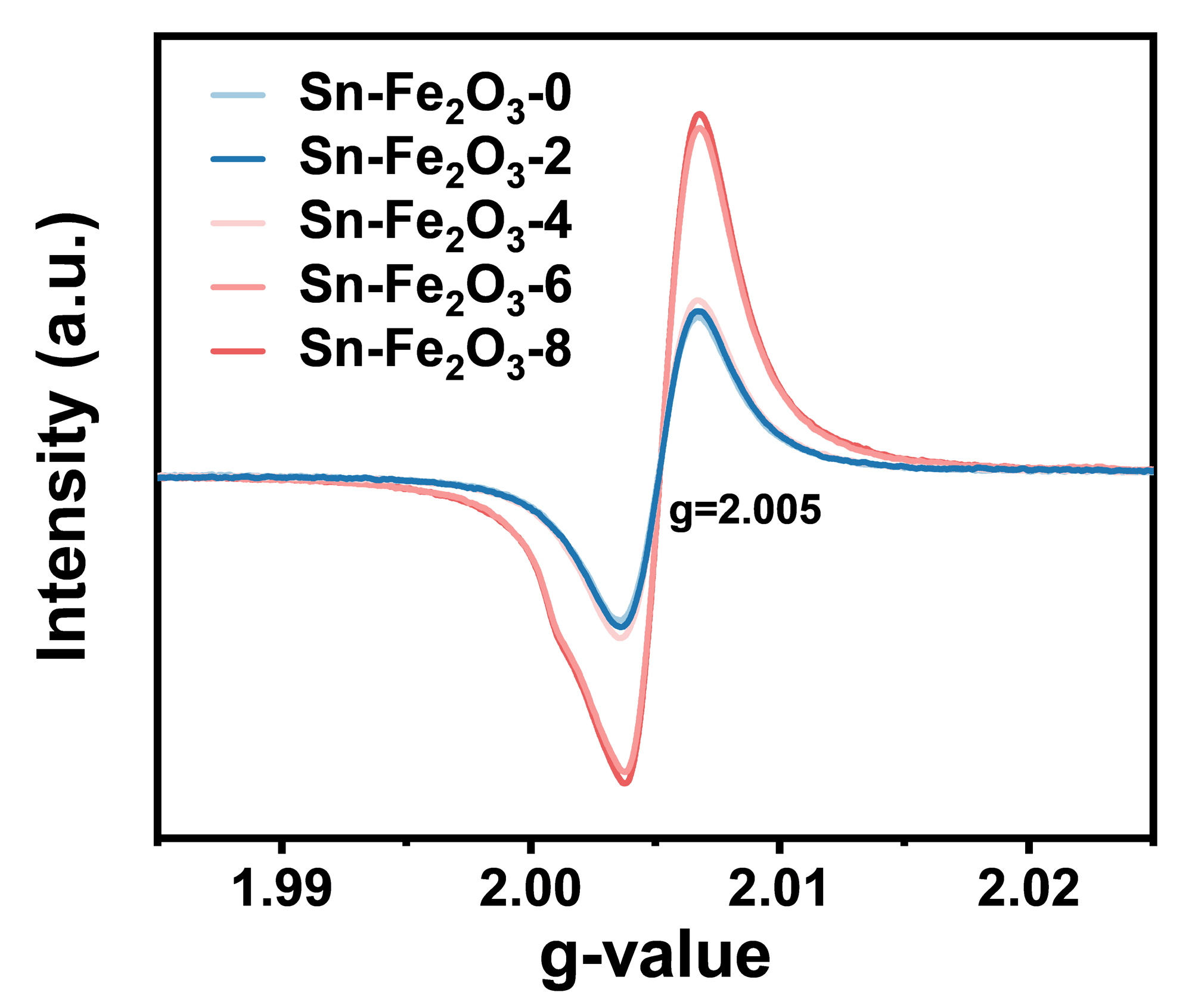


**Fig. S7** EPR spectrum of Sn-Fe_2_O_3_-0, Sn-Fe_2_O_3_-2, Sn-Fe_2_O_3_-4, Sn-Fe_2_O_3_-6 and Sn-Fe_2_O_3_-8


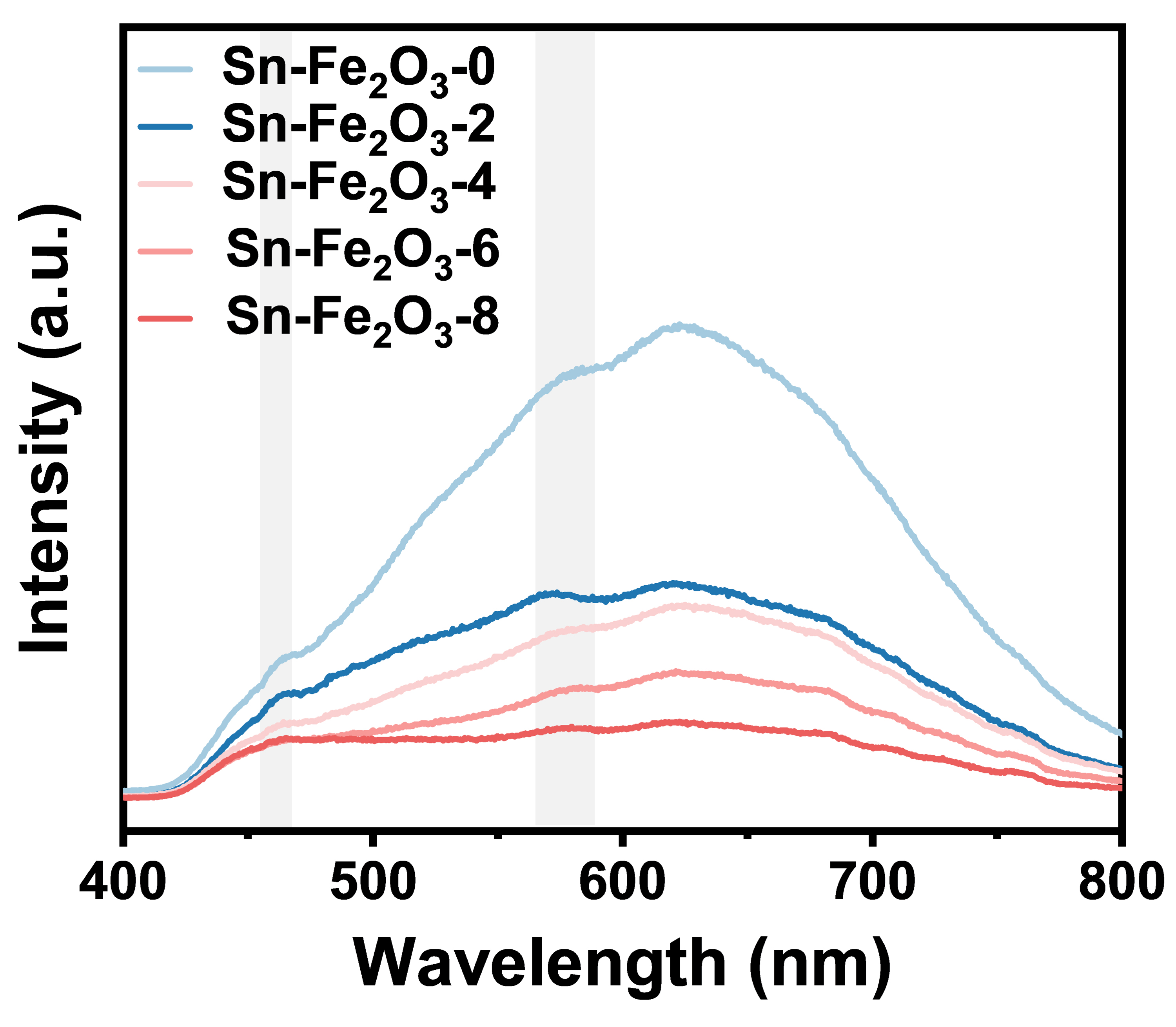


**Fig. S8** PL spectrum of Sn-Fe_2_O_3_-0, Sn-Fe_2_O_3_-2, Sn-Fe_2_O_3_-4, Sn-Fe_2_O_3_-6 and Sn-Fe_2_O_3_-8


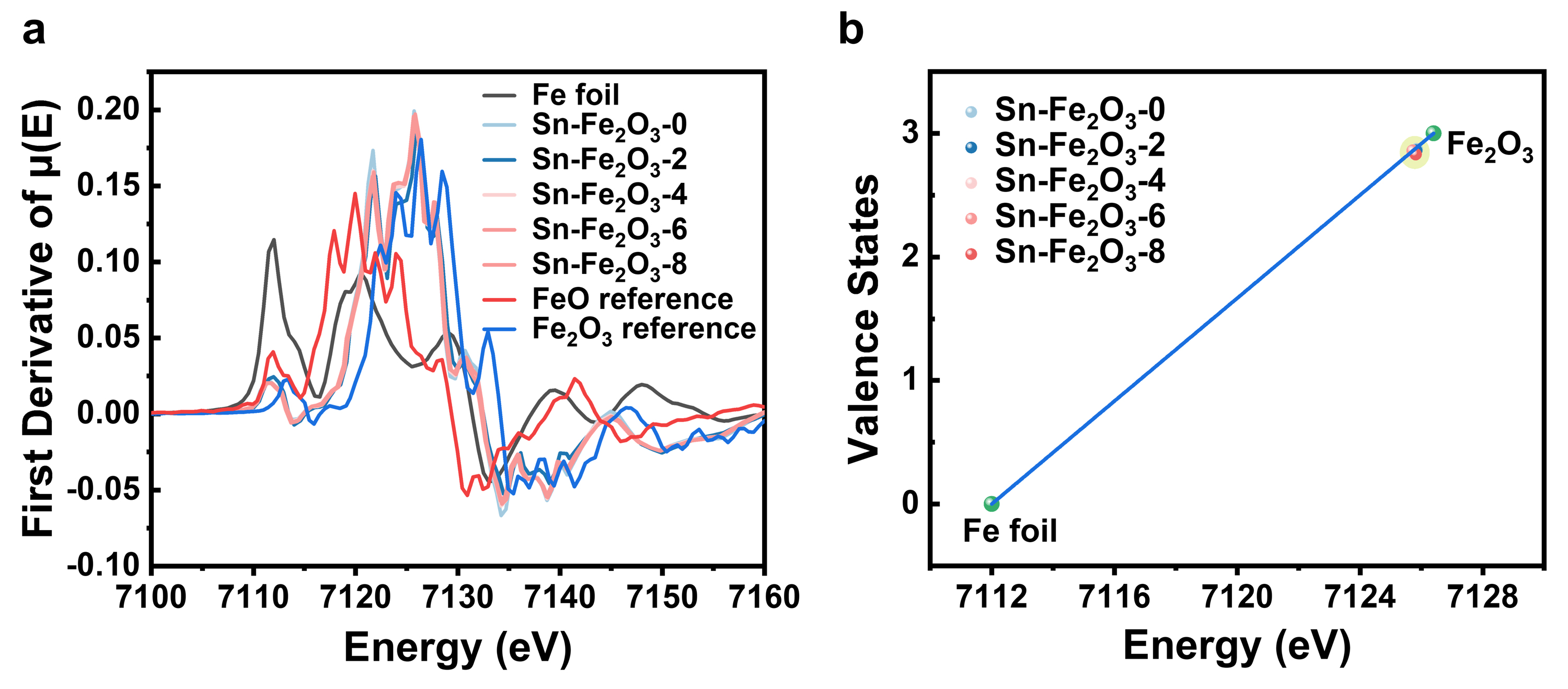


**Fig. S9** **(a)** First derivative of Fe *K*-edge XANES. **(b)** Fe average valence states determination in Sn-Fe_2_O_3_-X, using the Fe *K*-edge energy shift of the reference Fe foil and Fe_2_O_3_


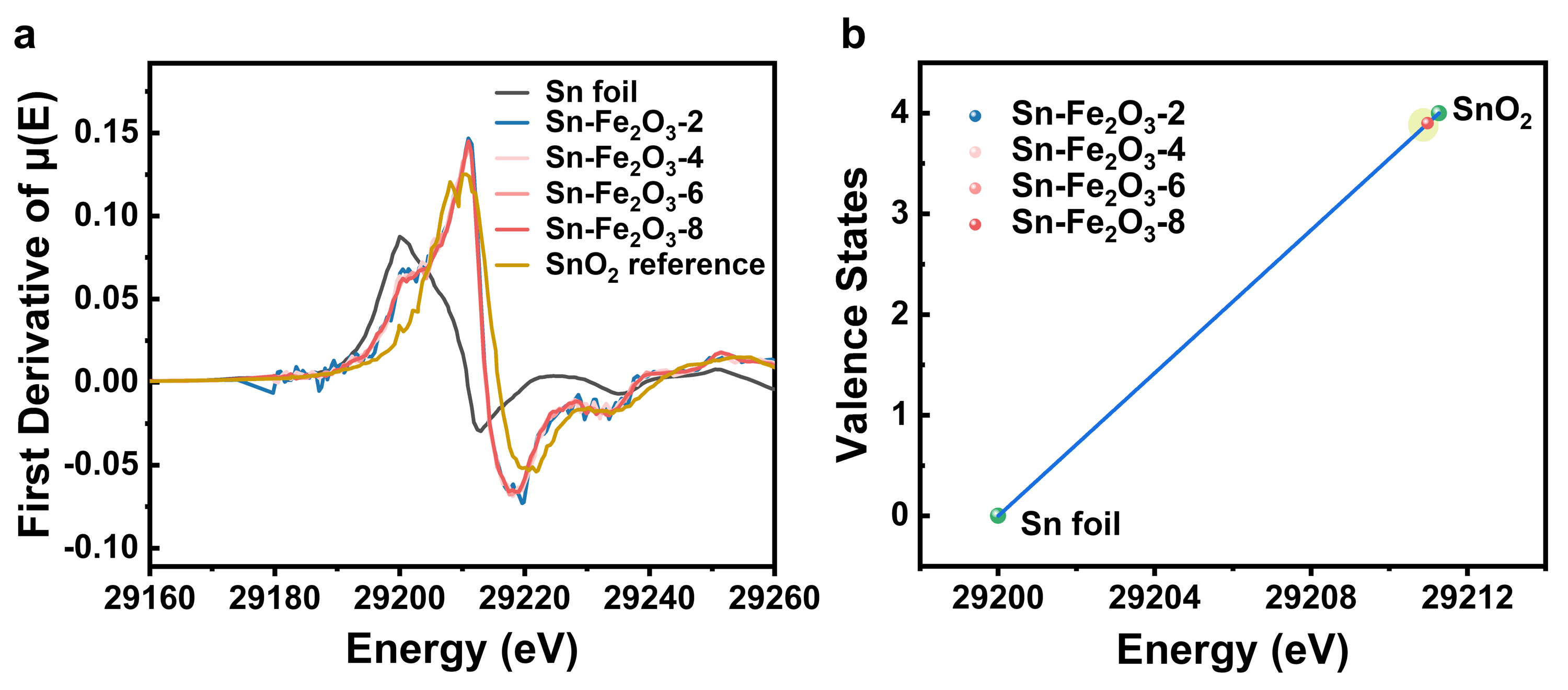


**Fig. S10** **(a)** First derivative of Sn *K*-edge XANES. **(b)** Sn average valence states determination in Sn-Fe_2_O_3_-X, using the Sn *K*-edge energy shift of the reference Sn foil and SnO_2_


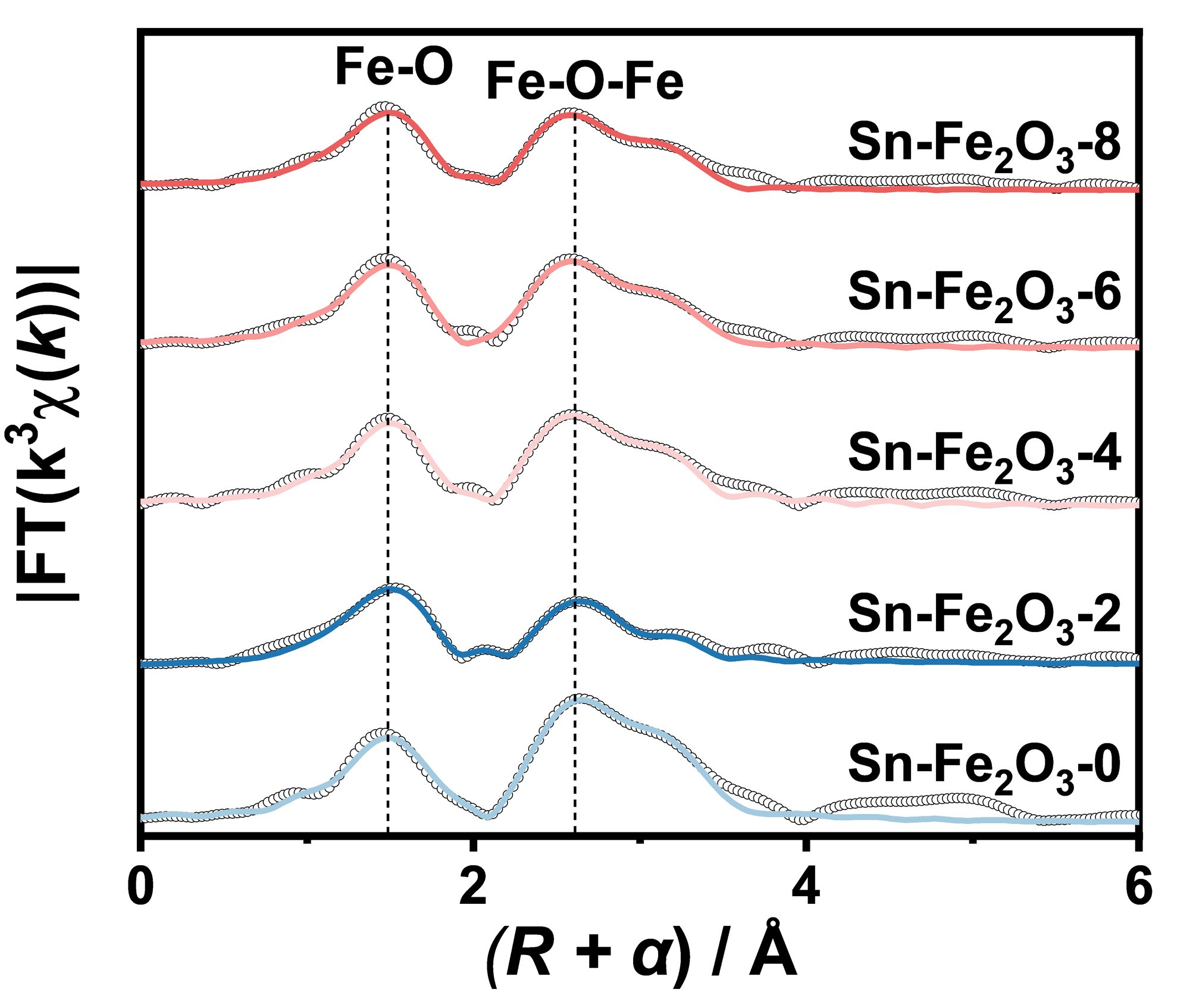


**Fig. S11** Fourier transforms of *k*^3^-weighted Fe *K*-edge EXAFS spectra (points) and representative fitting (line) in R-space of Sn-Fe_2_O_3_-0, Sn-Fe_2_O_3_-2, Sn-Fe_2_O_3_-4, Sn-Fe_2_O_3_-6, and Sn-Fe_2_O_3_-8

**
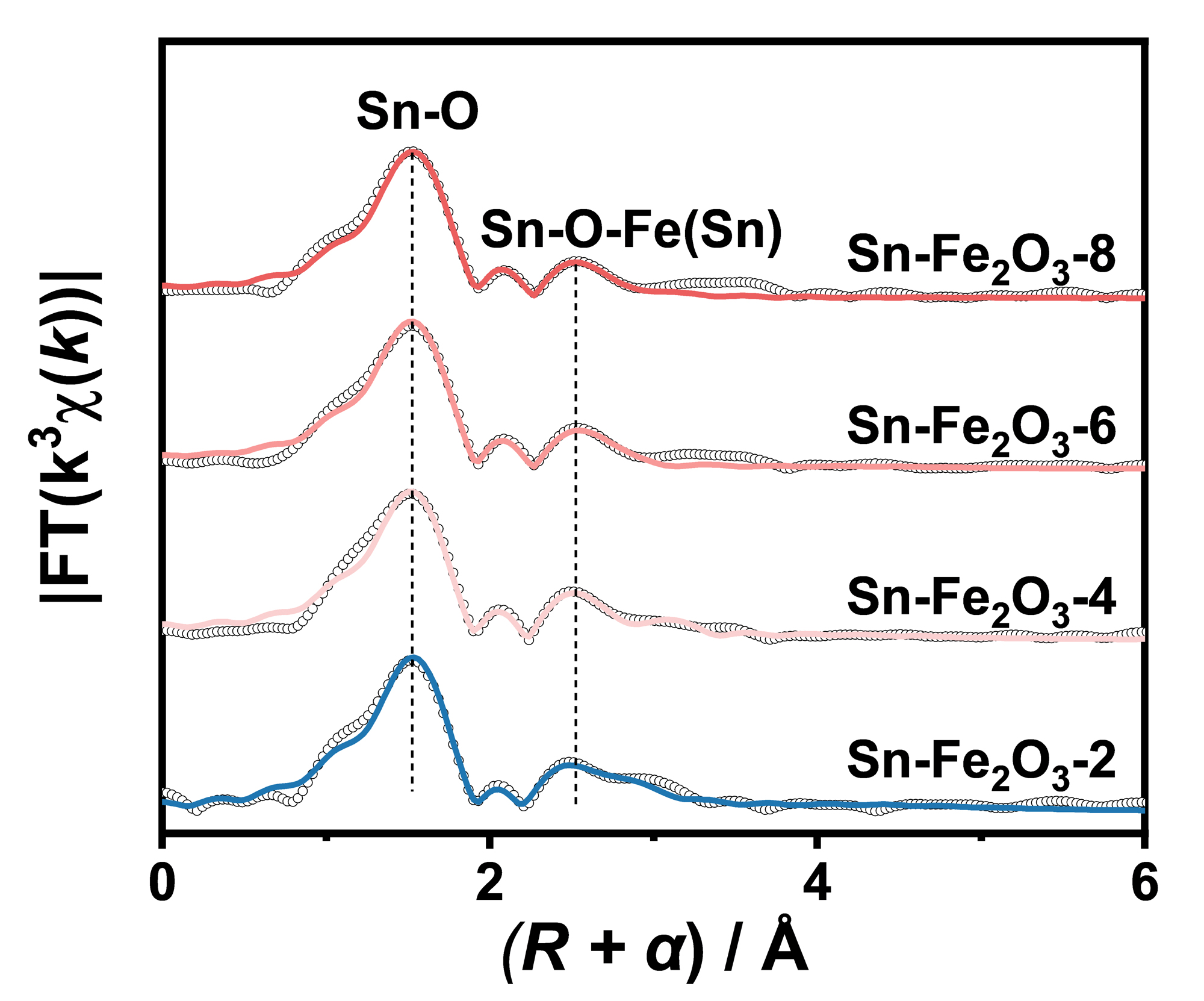
**

**Fig. S12** Fourier transforms of *k*3-weighted Sn *K*-edge EXAFS spectra (points) and representative fitting (line) in R-space of Sn-Fe_2_O_3_-2, Sn-Fe_2_O_3_-4, Sn-Fe_2_O_3_-6, and Sn-Fe_2_O_3_-8

**
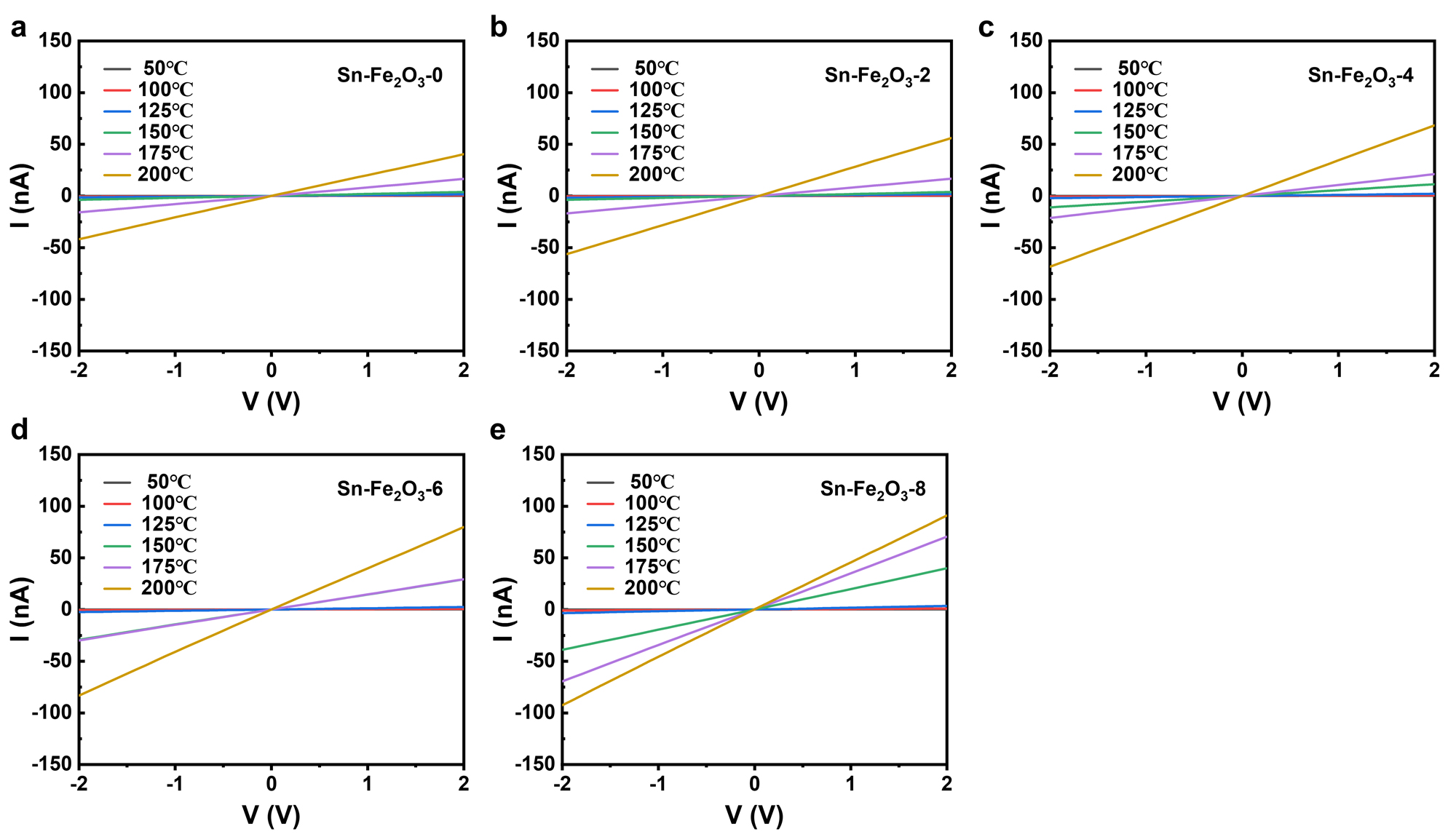
**

**Fig. S13** I-V curves of **(a)** Sn-Fe_2_O_3_-0, **(b)** Sn-Fe_2_O_3_-2, **(c)** Sn-Fe_2_O_3_-4, **(d)** Sn-Fe_2_O_3_-6, and **(e)** Sn-Fe_2_O_3_-8 under different temperatures


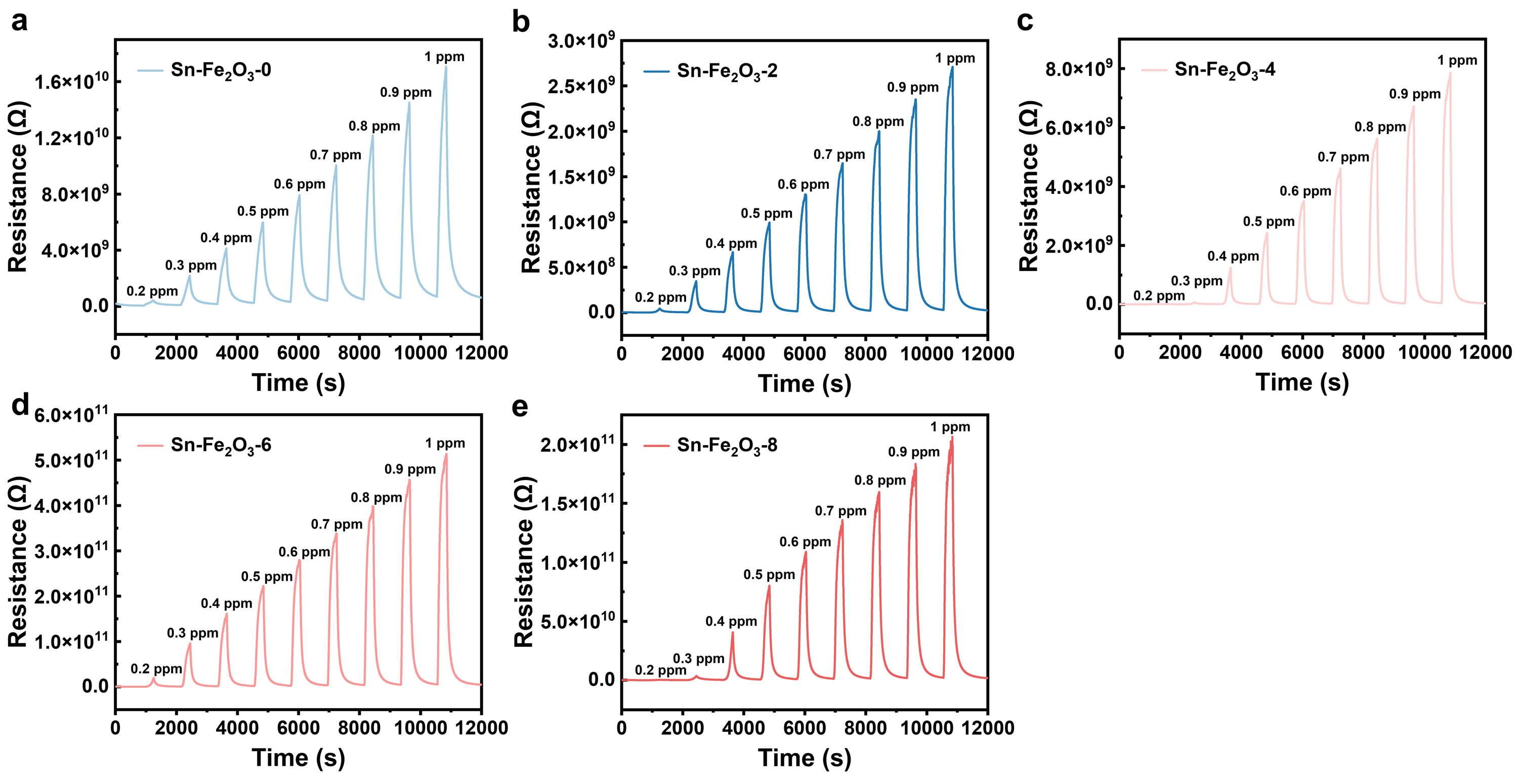


**Fig. S14** The dynamic resistance-time curves of **(a)** Sn-Fe_2_O_3_-0, **(b)** Sn-Fe_2_O_3_-2, **(c)** Sn-Fe_2_O_3_-4, **(d)** Sn-Fe_2_O_3_-6, **(e)** Sn-Fe_2_O_3_-8 with increasing NO_2_ concentration


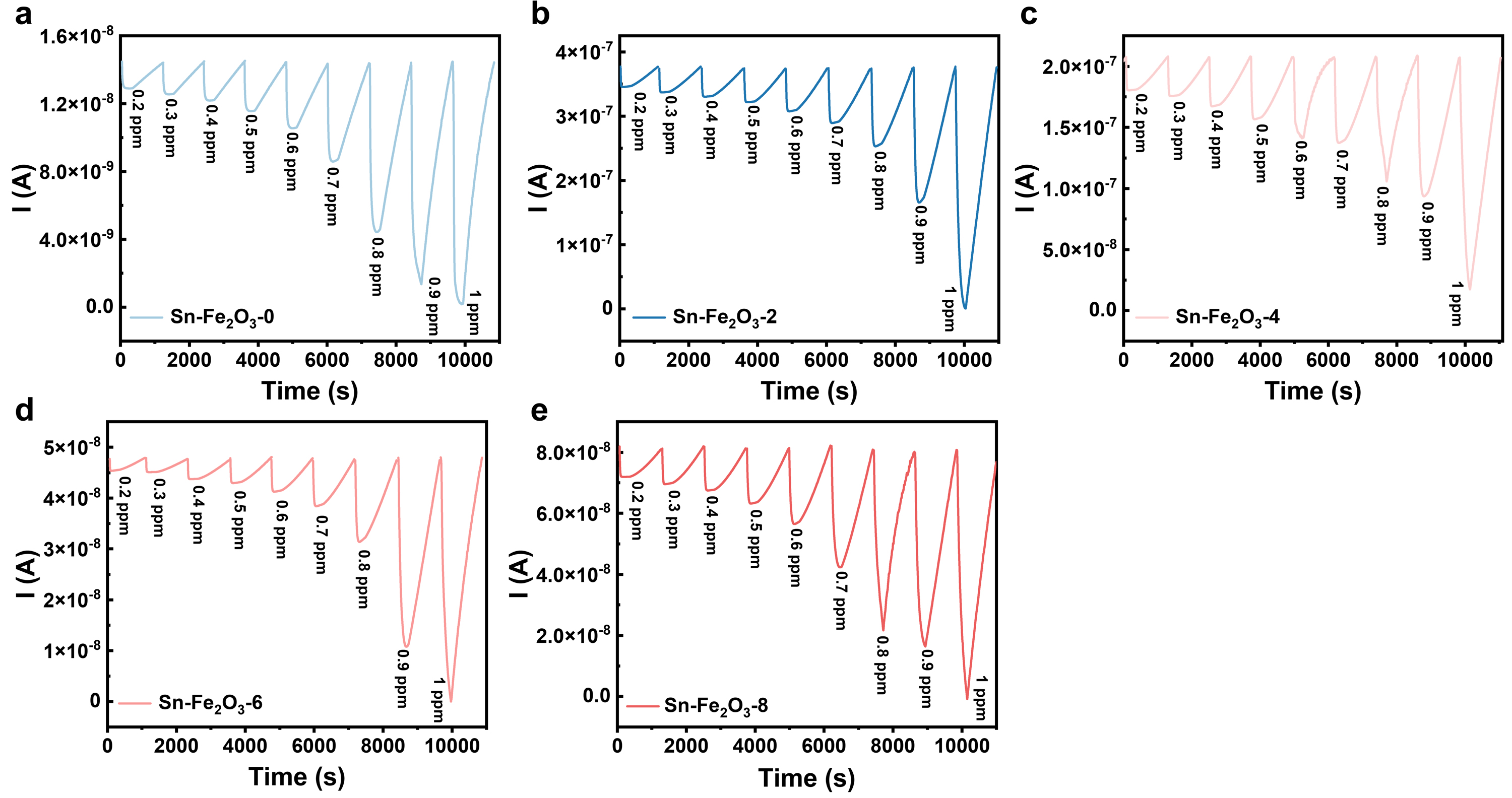


**Fig. S15** The dynamic current-time curves of **(a)** Sn-Fe_2_O_3_-0, **(b)** Sn-Fe_2_O_3_-2, **(c)** Sn-Fe_2_O_3_-4, **(d)** Sn-Fe_2_O_3_-6, **(e)** Sn-Fe_2_O_3_-8 with increasing NO_2_ concentration


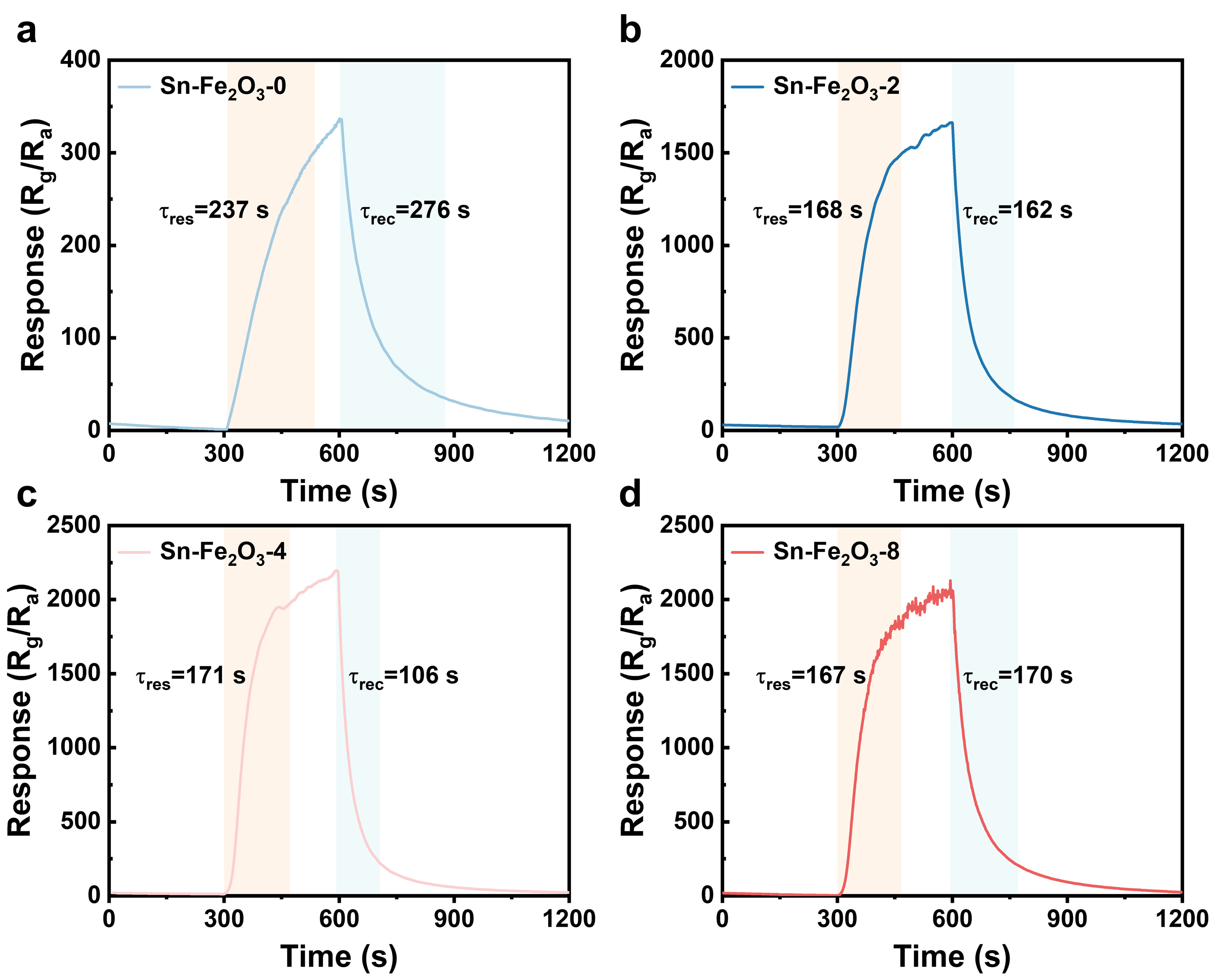


**Fig. S16** Response/recover curve of **a** Sn-Fe_2_O_3_-0, **b** Sn-Fe_2_O_3_-2, **c** Sn-Fe_2_O_3_-4, and **d** Sn-Fe_2_O_3_-8


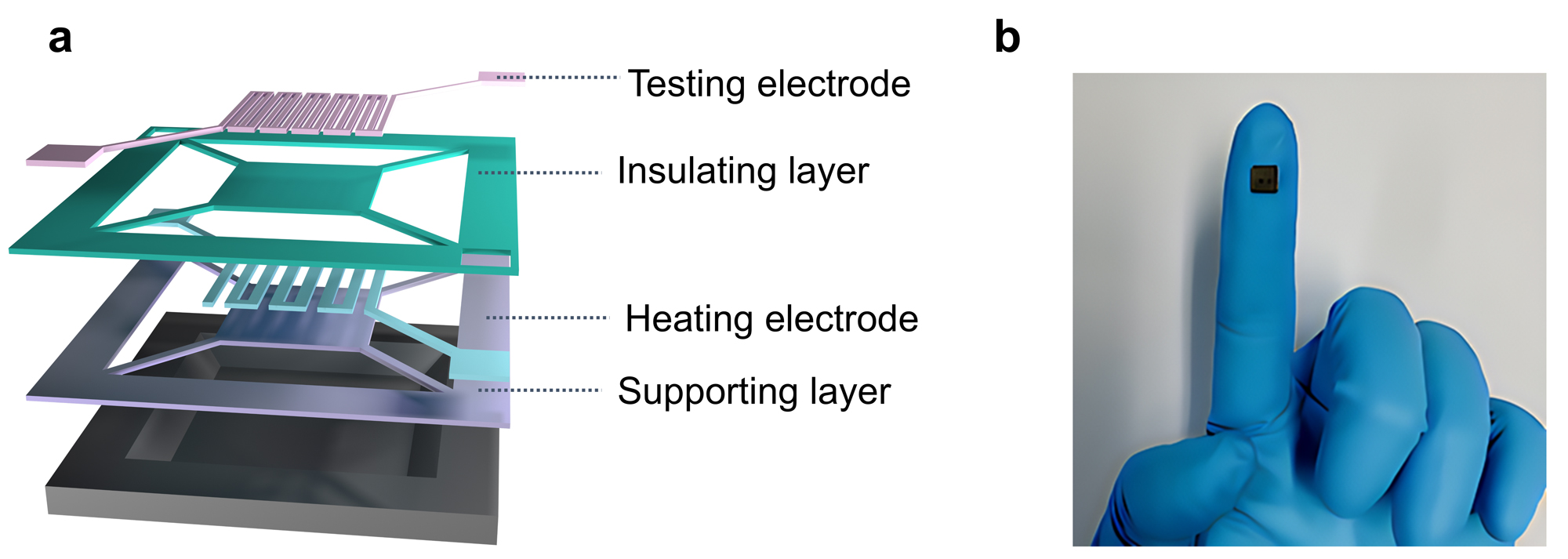


**Fig. S17** **a** structure diagram of MEMS hotplate, and **b** optical picture


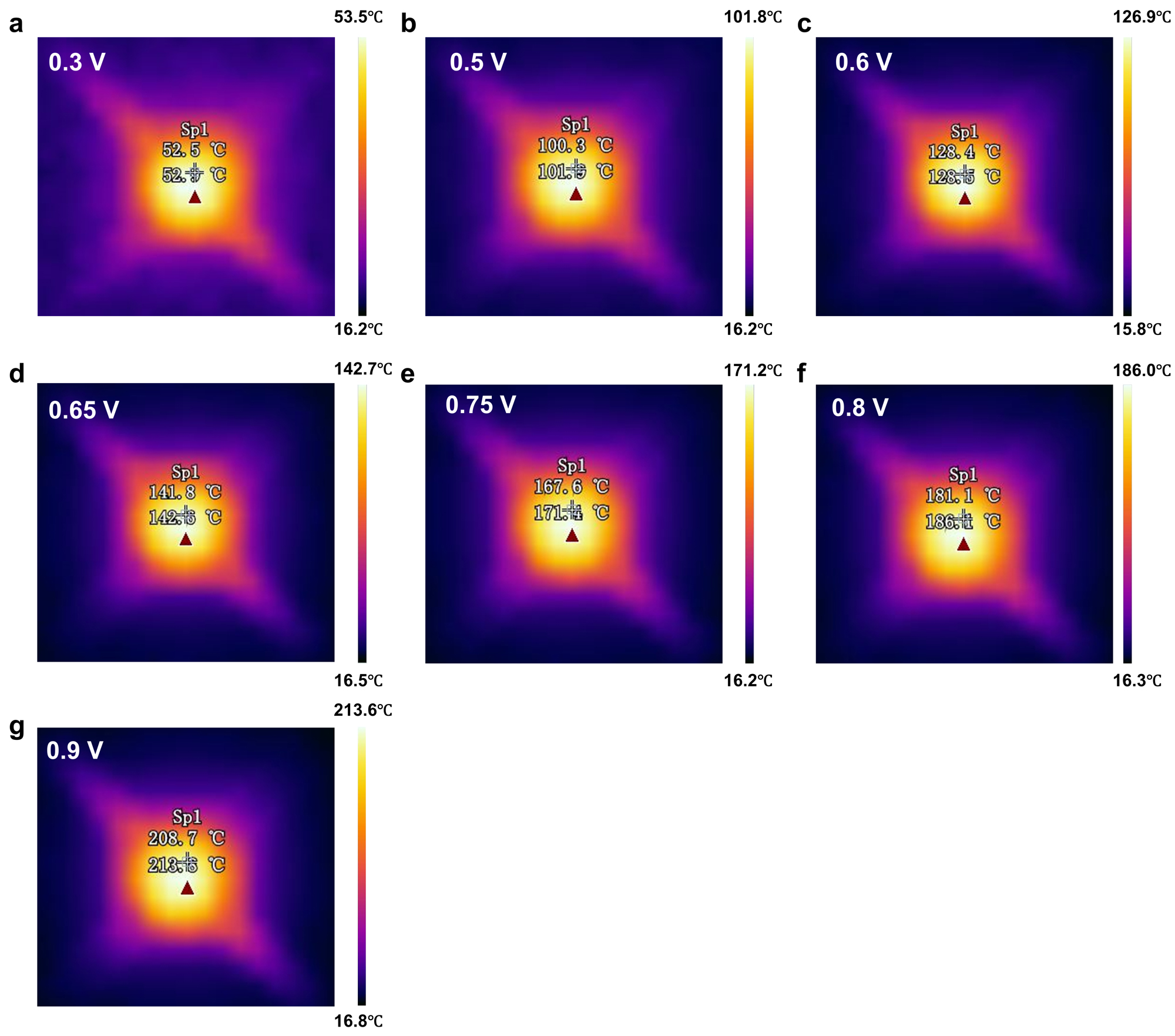


**Fig. S18** Infrared thermal map over the sensing area under heating voltage of **a** 0.3 V, **b** 0.5 V, **c** 0.6 V, **d** 0.65 V, **e** 0.75 V, **f** 0.8 V, **g** 0.9 V


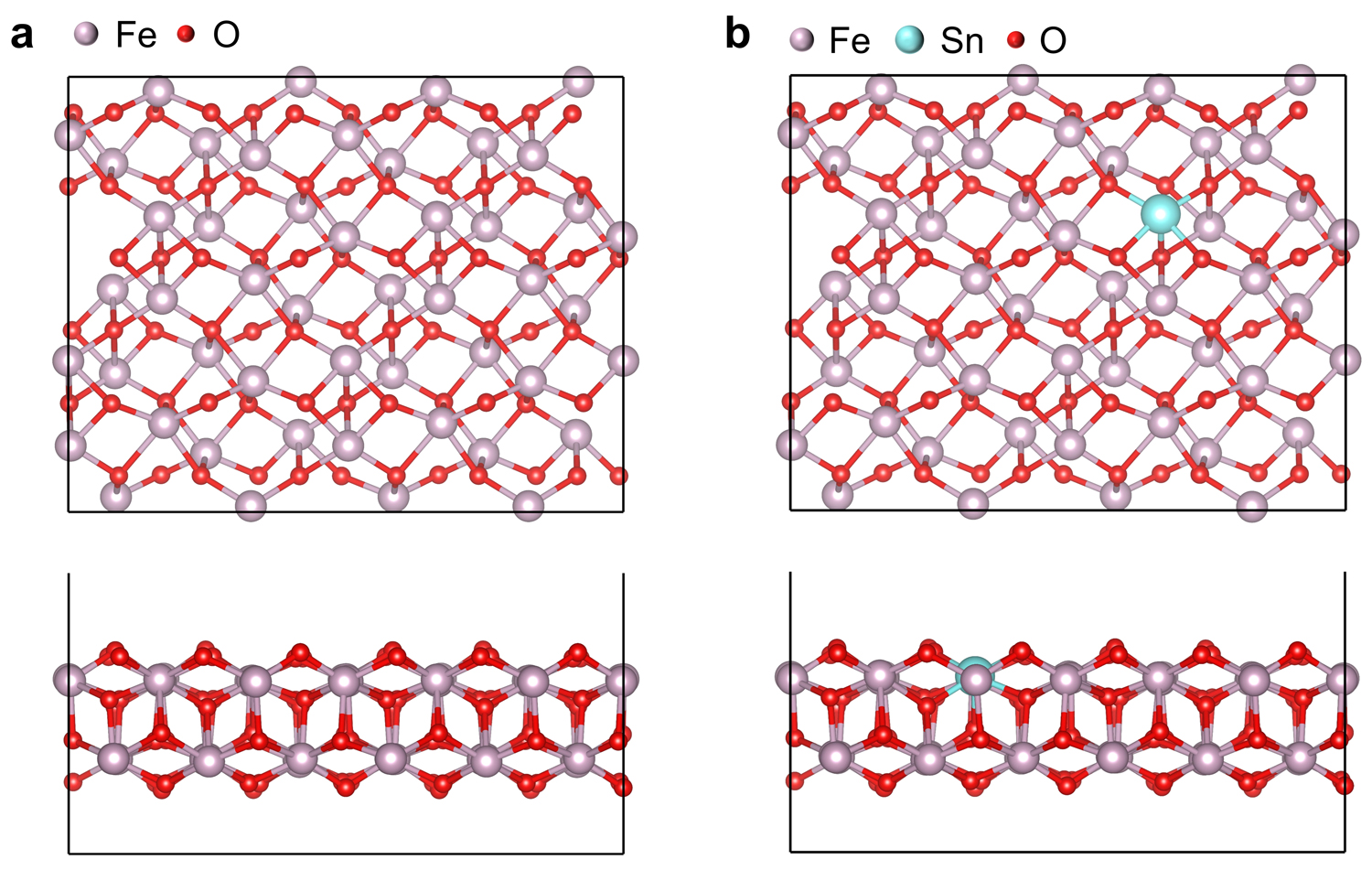


**Fig. S19** Top and side view of **a** Fe_2_O_3_(110) slab model, and **b** Fe_2_O_3_(110)-Sn slab model


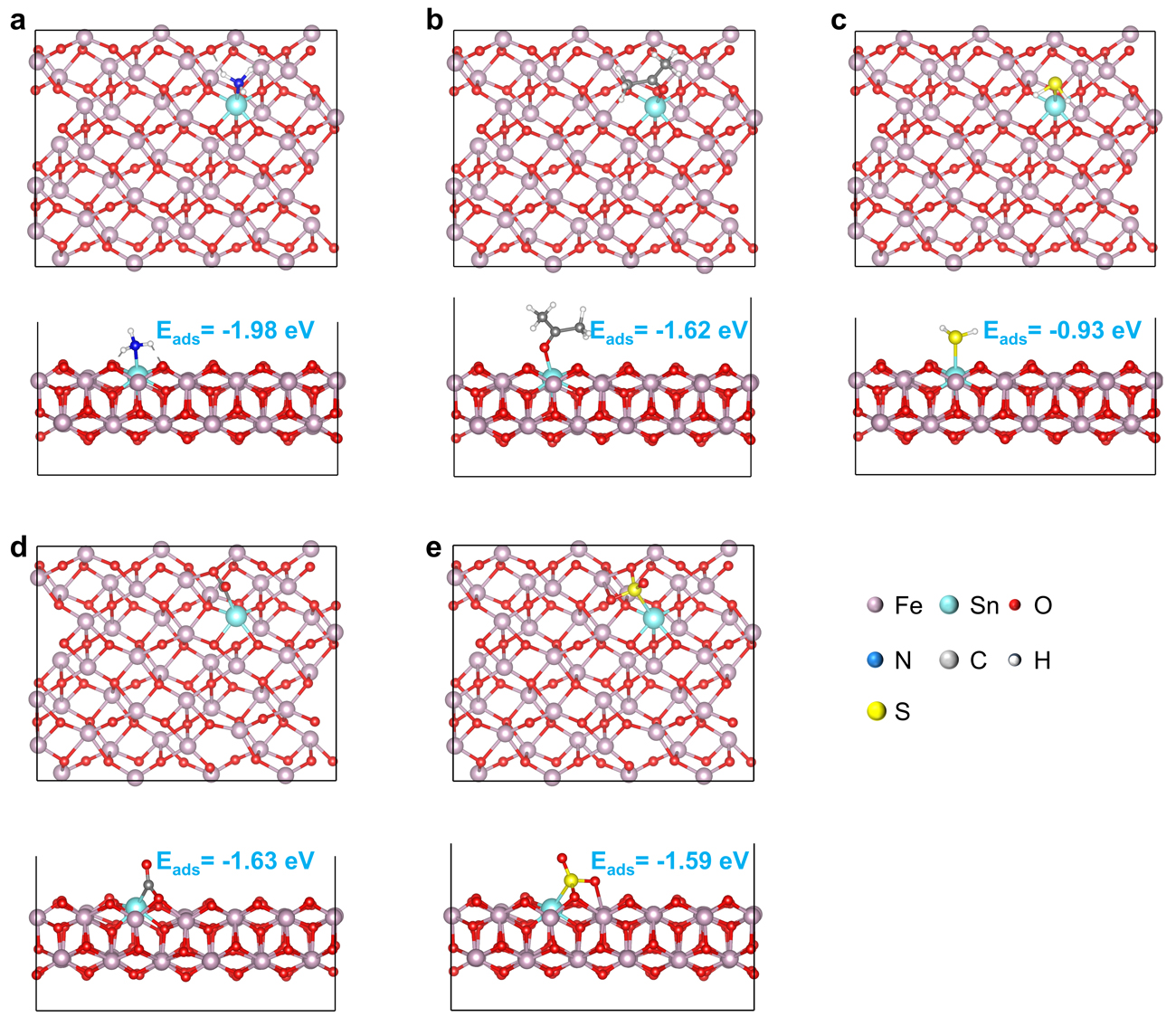


**Fig. S20** Top and side view of Fe_2_O_3_(110)-Sn adsorbing **a** NH_3_, **b** C_3_H_6_O, **c** H_2_S, d CO, and **e** H_2_S

**Table S1** Element content in Sn-Fe_2_O_3_-X determined by ICP-OES

| **Samples** | **Ion concentration (mg/L)** | | **Atomic ratio** |
| --- | --- | --- | --- |
|  | Sn | Fe | Sn/Fe |
| Sn-Fe_2_O_3_-0 | **\** | **\** | **\** |
| Sn-Fe_2_O_3_-2 | 2.415 | 206.049 | 1.2% |
| Sn-Fe_2_O_3_-4 | 6.438 | 168.795 | 3.8% |
| Sn-Fe_2_O_3_-6 | 15.537 | 250.727 | 5.8% |
| Sn-Fe_2_O_3_-8 | 17.791 | 241.833 | 7.4% |

**Table S2** Valence states of Fe, O species over Sn-Fe_2_O_3_-X samples derived from XPS analysis

|  | **Fe species (%)** | | **O species (%)** | | |
| --- | --- | --- | --- | --- | --- |
|  | **Fe^2+^** | **Fe^3+^** | **O_L_** | **O_V_** | **O_C_** |
| Sn-Fe_2_O_3_-0 | 27.3% | 72.7% | 77.5 | 16.1 | 6.4 |
| Sn-Fe_2_O_3_-2 | 29.8% | 70.2% | 77.7 | 18.2 | 4.1 |
| Sn-Fe_2_O_3_-4 | 34.6% | 65.4% | 74.3 | 19.1 | 6.6 |
| Sn-Fe_2_O_3_-6 | 45.1% | 54.9% | 71.8 | 20.2 | 8.0 |
| Sn-Fe_2_O_3_-8 | 41.1% | 58.9% | 75.4 | 20.4 | 4.2 |

**Table S3** Fitting results for the Fe *K*-edge EXAFS data for the Sn-Fe_2_O_3_-X

| **Sample** | **N_Fe-O_** | **R_Fe-O_ (Å)** | **σ^2^ (Å^2^)** | **ΔE_0_ (eV)** | **R_factor_** |
| --- | --- | --- | --- | --- | --- |
| Sn-Fe_2_O_3_-0 | 5.4±0.7 | 1.986±0.006 | 0.0130±0.0030 | 1.20±1.3 | 0.0050 |
| Sn-Fe_2_O_3_-2 | 5.2±0.3 | 1.962±0.018 | 0.0120±0.0014 | 2.94±0.5 | 0.0018 |
| Sn-Fe_2_O_3_-4 | 5.0±0.7 | 1.978±0.002 | 0.0124±0.0030 | 0.83±1.3 | 0.0091 |
| Sn-Fe_2_O_3_-6 | 5.1±0.7 | 1.974±0.006 | 0.0120±0.0027 | 0.63±1.3 | 0.0075 |
| Sn-Fe_2_O_3_-8 | 5.0±0.6 | 1.972±0.008 | 0.0130±0.0024 | 0.47±1.1 | 0.0047 |

**Note:** N, coordination number; R, bonding distance; σ^2^, Debye-Waller factor; ΔE_0_, inner potential correction; R_factor_, difference between modeled and experimental data.

**Table S4** Fitting results for the Sn *K*-edge EXAFS data for the Sn-Fe_2_O_3_-X

| **Sample** | **N_Sn-O_** | **R_Sn-O_ (Å)** | **σ^2^ (Å^2^)** | **ΔE_0_ (eV)** | **R_factor_** |
| --- | --- | --- | --- | --- | --- |
| Sn-Fe_2_O_3_-2 | 5.3±0.9 | 2.018±0.04 | 0.0026±0.0025 | 5.72±2.2 | 0.0172 |
| Sn-Fe_2_O_3_-4 | 5.3±0.9 | 2.05±0.07 | 0.0036±0.0029 | 1.72±2.3 | 0.0271 |
| Sn-Fe_2_O_3_-6 | 5.3±0.8 | 2.06±0.08 | 0.003±0.0024 | 2.02±1.89 | 0.0131 |
| Sn-Fe_2_O_3_-8 | 5.3±0.6 | 2 .06±0.08 | 0.003±0.0019 | 2.73±1.50 | 0.0065 |

**Note:** N, coordination number; R, bonding distance; σ^2^, Debye-Waller factor; ΔE_0_, inner potential correction; R_factor_, difference between modeled and experimental data.

**Table S5** Response/recovery times of Sn-Fe_2_O_3_-X to 1 ppm NO_2_

| **Samples** | **𝜏_res_ (s)** | **𝜏_res_ (s)** |
| --- | --- | --- |
| Sn-Fe_2_O_3_-0 | 237 | 276 |
| Sn-Fe_2_O_3_-2 | 168 | 162 |
| Sn-Fe_2_O_3_-4 | 171 | 106 |
| Sn-Fe_2_O_3_-6 | 162 | 148 |
| Sn-Fe_2_O_3_-8 | 167 | 170 |

**Table S6** Comparison between NO_2_ gas sensing properties of this work with other reported literatures

| Material | NO_2_ conc.  (ppm) | T  (℃) | Response (R_g_/R_a_) | 𝜏_res_/𝜏_recov_  (s) | LOD  (ppb) | Refs. |
| --- | --- | --- | --- | --- | --- | --- |
| Se-Fe_2_O_3_ | 5 | 130 | 5.4 | 7/14 | 27 | [41] |
| Ag/Fe_2_O_3_ | 1 | 150 | 2.4 | \ | 20 | [42] |
| α-Fe_2_O_3_/Co_3_O_4_/rGO | 2 | 130 | 17.64 | 44/50 | 280 | [43] |
| α-Fe_2_O_3_/BiVO_4_ | 2 | 110 | 7.8 | \ | \ | [44] |
| rGO/α-Fe_2_O_3_ | 5 | RT | 8.2 | 126/2400 | 50 | [45] |
| α-Fe_2_O_3_-ZnO | 10 | 200 | 6.34 | 26/185 | \ | [46] |
| WSe_2_ | 1 | RT  (UV) | 9 | 53/90 | 68 | [47] |
| In_2_O_3_/ZnO | 1 | RT  (UV) | 6 | 36/68 | 50 | [48] |
| rGO/Bi_2_S_3_ | 1 | RT | 9.8 | 22/106 | 25 | [49] |
| Sn-Fe_2_O_3_-6 | 1 | 150 | 2646.6 | 162/148 | 10 | This work |

**Table S7** Adsorption energy of NO_2_ on the surface of the Fe_2_O_3_(110) and Sn-Fe_2_O_3_(110) surface

|  | **Energy** | **Adsorption energy (eV)** |
| --- | --- | --- |
| Fe_2_O_3_(110) | -792.312620 | -0.48 |
| Fe_2_O_3_(110)-NO_2_ | -811.166990 |  |
| Fe_2_O_3_(110)-Sn | -791.412460 | -2.20 |
| Fe_2_O_3_(110)-Sn-NO_2_ | -811.988240 |  |
| NO_2_ | -18.38 |  |

**Table S8** Adsorption energies of different gases on the optimum structure of Fe_2_O_3_(110)-Sn

| **Gas** | **E_ads_ (eV)** |
| --- | --- |
| Nitrogen dioxide (NO_2_) | -2.20 |
| Ammonia (NH_3_) | -1.98 |
| Carbon monoxide (CO) | -1.63 |
| Acetone (C_3_H_6_O) | -1.62 |
| Sulfur dioxide (SO_2_) | -1.59 |
| Hydrogen sulfide (H_2_S) | -0.93 |
